# Cardiac MR: From Theory to Practice

**DOI:** 10.3389/fcvm.2022.826283

**Published:** 2022-03-03

**Authors:** Tevfik F. Ismail, Wendy Strugnell, Chiara Coletti, Maša Božić-Iven, Sebastian Weingärtner, Kerstin Hammernik, Teresa Correia, Thomas Küstner

**Affiliations:** ^1^School of Biomedical Engineering and Imaging Sciences, King's College London, London, United Kingdom; ^2^Cardiology Department, Guy's and St Thomas' Hospital, London, United Kingdom; ^3^Queensland X-Ray, Mater Hospital Brisbane, Brisbane, QLD, Australia; ^4^Magnetic Resonance Systems Lab, Delft University of Technology, Delft, Netherlands; ^5^Computer Assisted Clinical Medicine, Heidelberg University, Mannheim, Germany; ^6^Lab for AI in Medicine, Technical University of Munich, Munich, Germany; ^7^Department of Computing, Imperial College London, London, United Kingdom; ^8^Centre of Marine Sciences, Faro, Portugal; ^9^Medical Image and Data Analysis (MIDAS.lab), Department of Diagnostic and Interventional Radiology, University Hospital of Tübingen, Tübingen, Germany

**Keywords:** cardiovascular MR, deep learning, CMR protocol, quantitative imaging, image reconstruction, sequence design, imaging acceleration, image processing

## Abstract

Cardiovascular disease (CVD) is the leading single cause of morbidity and mortality, causing over 17. 9 million deaths worldwide per year with associated costs of over $800 billion. Improving prevention, diagnosis, and treatment of CVD is therefore a global priority. Cardiovascular magnetic resonance (CMR) has emerged as a clinically important technique for the assessment of cardiovascular anatomy, function, perfusion, and viability. However, diversity and complexity of imaging, reconstruction and analysis methods pose some limitations to the widespread use of CMR. Especially in view of recent developments in the field of machine learning that provide novel solutions to address existing problems, it is necessary to bridge the gap between the clinical and scientific communities. This review covers five essential aspects of CMR to provide a comprehensive overview ranging from CVDs to CMR pulse sequence design, acquisition protocols, motion handling, image reconstruction and quantitative analysis of the obtained data. (1) The basic MR physics of CMR is introduced. Basic pulse sequence building blocks that are commonly used in CMR imaging are presented. Sequences containing these building blocks are formed for parametric mapping and functional imaging techniques. Commonly perceived artifacts and potential countermeasures are discussed for these methods. (2) CMR methods for identifying CVDs are illustrated. Basic anatomy and functional processes are described to understand the cardiac pathologies and how they can be captured by CMR imaging. (3) The planning and conduct of a complete CMR exam which is targeted for the respective pathology is shown. Building blocks are illustrated to create an efficient and patient-centered workflow. Further strategies to cope with challenging patients are discussed. (4) Imaging acceleration and reconstruction techniques are presented that enable acquisition of spatial, temporal, and parametric dynamics of the cardiac cycle. The handling of respiratory and cardiac motion strategies as well as their integration into the reconstruction processes is showcased. (5) Recent advances on deep learning-based reconstructions for this purpose are summarized. Furthermore, an overview of novel deep learning image segmentation and analysis methods is provided with a focus on automatic, fast and reliable extraction of biomarkers and parameters of clinical relevance.

## Introduction

Over the past 40 years, cardiovascular magnetic resonance (CMR) has evolved from an esoteric research tool found in the confines of large academic supraregional tertiary referral centers to being an indispensable clinical tool that routinely changes patient management across the breadth of modern cardiovascular practice (1). Increasing clinical recognition of the transformative role this technology can play in patient care has led to its growing availability in secondary care settings too, although significant barriers remain to its greater adoption world-wide, particularly in Africa.

CMR is a versatile non-invasive and radiation-free imaging modality that provides a comprehensive assessment of multiple parameters of cardiac function and anatomy in a single examination. CMR plays a major role in the diagnosis and management of cardiovascular disease. However, aside from cost, there remain major obstacles for the widespread usage of this technique like: (i) complex underlying physics and technology, (ii) data analysis and interpretation, (iii) large number of pulse sequences and parameters to choose from, (iv) challenges from the inherent cardiac and respiratory motion, and (v) duration of examination. The recent hype around artificial intelligence algorithms designed to overcome these hurdles has raised new questions around the reliability, accuracy, and stability of this technology. Therefore, to help shape the future of CMR, it is essential to bridge the gap between theory and practice, and thus, to promote a bridge of scientific knowledge between the research and clinical communities by improving (maintaining or updating) their knowledge of CMR technical principles and clinical applications.

This review provides an overview of five essential aspects of CMR which have been covered separately in-depth in other review papers (2–11). We address: (1) data acquisition sequences and common artifacts, (2) clinical applications, (3) clinical examination protocols, (4) image acceleration, reconstruction, and motion handling, (5) artificial intelligence-assisted reconstruction and analysis. In addition, this review provides hands-on tutorials and videos that can be found at ismrm-mit-cmr.github.io. More specifically, Section The Physics Behind Cardiovascular MR describes the key physical principles of CMR, most common pulse sequences and preparation pulses, and the physics behind the most common artifacts. Section Clinical Cardiovascular MR: What do we See and Why do we Need it? covers the clinical application of CMR in the diagnosis of a spectrum of cardiovascular diseases. Section Clinical Cardiovascular MR: How Should we Perform the Examination describes how to complete a comprehensive examination and deal with challenging patients. Section CMR Image Quality: No Free Lunch provides an overview of scan acceleration acquisition and image reconstruction methods while also describing current solutions to overcome challenges from cardiac and respiratory motion. Finally, Section Artificial Intelligence for Cardiovascular MR describes machine learning methods used for automated quantitative analysis of CMR data.

## The Physics Behind Cardiovascular MR

In this section we aim to provide a brief overview of the physical principles and basic mathematical concepts behind magnetic resonance imaging (MRI) targeted to create the necessary background to understand modern CMR methods. This section will give an overview of the physics of nuclear magnetic resonance and relaxation, essential for describing the concepts behind image formation and the k-space formalism. Furthermore, basic building blocks of MRI are introduced, and common cardiac MR sequences are described.

### Magnetization Formation and Dynamics

MRI is based on a magnetic property that is intrinsic to certain nuclei, some of which can be found all throughout the human body. Nuclei [and (sub)atomic particles] possess an intrinsic quantum mechanical property called spin. Mathematically the spin can be described as the angular momentum of a spinning sphere. As a quantum mechanical quantity, however, the spin can only have a discrete set of states. By convention, the number of spin states are described according to the spin quantum number *S* with integer or half-integer values, giving rise to 2*S*+1 different spin states. In MRI, the nucleus of greatest importance can be found in hydrogen atoms (^1^H): It comprises only a single proton with $S\;=\;\frac{1}{2}\;$and, thus, two spin states. These are commonly denoted as +½ (“spin-up”) and –½ (“spin-down”). Due to the classical relationship between angular momentum and magnetic moment of a rotating charged particle, the spin *S* is always associated with a magnetic moment μ via the particle-specific gyromagnetic ratio γ ([rad/sT]):


(1) $$\begin{array}{l} \mu =\gamma S. \end{array}$$

In a proton ensemble the magnetic moments of the nuclei are randomly orientated unless an external magnetic field *B*_0_ is applied. In this case, all particles will align depending on their magnetic moment either parallel (“spin-up”) or anti-parallel (“spin-down”) to the applied field. Now, spins parallel to the magnetic field are in a lower energy state compared with those in the opposite direction. Hence, the energy levels of the spin states are separated by Δ*E* = γℏ*B*_0_, with reduced Planck constant ℏ. This is also known as the Zeeman effect. Due to the angular momentum, the magnetic moment is also associated with a precession around ${\vec{B}}_{0}$. The rotational frequency of this precession is called the Larmor frequency ω_*L*_:


(2) $$\begin{array}{l} \omega_{L}=\gamma \;B_{0} \end{array}$$

For clinical MRI field strengths (0.5T−7T), this frequency is usually found in the radio frequency (RF) range. At thermal equilibrium, there is a slight excess of protons in the “spin-up” state due to its lower energy. Thus, the net magnetization $\vec{M}$ averaged over all protons will be oriented along and precess around ${\vec{B}}_{0}$. Following the correspondence principle, this net magnetization $\vec{M}$ and its precession motion can be described with classical mechanics, where the precession dynamics resemble those of a spinning top. The net magnetization $\vec{M}$ can be perturbed if protons are excited from the thermal equilibrium. In the analogy of the spinning top, this would mean tilting its rotation axis to the side. To achieve this, a so-called RF pulse that produces a resonant magnetic field ${\vec{B}}_{1}$ oscillating at ω_*L*_ needs to be applied. During this RF pulse, energy will be deposited in the spin system and some of the protons will flip to the “spin-down” state. Depending on the duration and strength of the RF pulse, the direction of $\vec{M}$ progressively tips away from ${\vec{B}}_{0}$ leading to a transverse component perpendicular to ${\vec{B}}_{0}$. Thereby, the polar angle α between $\vec{M}$ and ${\vec{B}}_{0}\;$is referred to as flip angle. Assuming that the initial magnetic field ${\vec{B}}_{0}\;$is along the *z*-axis, then the transverse and longitudinal parts of $\vec{M}$ are denoted as ${\vec{M}}_{xy}$ and ${\vec{M}}_{z}$, respectively. The above-described phenomenon is called nuclear magnetic resonance and gives MR imaging its name as the underlying physical principle.

### MR Signal and Relaxation: Time to Relax

The precession of $\vec{M}$ leads to an oscillating magnetic field. We can picture the precessing magnetization as a rotating bar magnet in classical mechanics. This can be detected using a nearby coil where the time-varying magnetic flux induces a measurable electric current via the Faraday-Lenz principle. After the RF pulse has been turned off, the net magnetization continues to precess around ${\vec{B}}_{0}$. However, over time, the energy transferred to the system dissipates and the magnetization recovers to the thermal equilibrium state ${\vec{M}}_{0}$. This process is known as longitudinal relaxation and can be described by an exponential growth function with characteristic time constant *T*_1_:


(3) $$\begin{array}{l} M_{z}\left(t\right)=\;M_{z}\left(0\right)-\;\left(M_{z}\left(0\right)-M_{z,0}\right)e^{-\frac{t}{T_{1}}}. \end{array}$$

Here, *M*_*z*_(0) = *M*_*z*_(*t* = 0) is the flip angle dependent initial magnetization, and *M*_*z*, 0_ the longitudinal magnetization at thermal equilibrium.

Besides the regrowth of ${\vec{M}}_{z}$, the transverse magnetization is subject to an additional relaxation process: the transverse component ${\vec{M}}_{xy}$ is only preserved if all spins precess with the same frequency, i.e., point to the same direction. But, due to differences in the microscopic environment, each spin experiences slightly different magnetic fields. As a result, individual spins precess with slightly different frequencies. Over time, this leads to a dephasing of the spins and to a decrease of ${\vec{M}}_{xy}$. This is referred to as transverse relaxation and can be modeled by an exponential decay with characteristic decay time *T*_2_:


(4) $$\begin{array}{l} M_{xy}\left(t\right)=\;M_{xy,0}e^{-\frac{t}{T_{2}}}, \end{array}$$

where *M*_*xy*,0_ describes the transverse magnetization after excitation. In addition, inhomogeneity of the main magnetic field (Δ*B*_0,*i*_) accelerates dephasing and leads to an effective decay time denoted as $T_{2}^{*}$: $\frac{1}{T_{2}^{*}}=\frac{1}{T_{2}}+\gamma \Delta B_{0,i}.\;$Thus, the actually observed decay time $T_{2}^{*}$ is always equal to or shorter than *T*_2_ and usually shorter than *T*_1_. Both relaxation processes are influenced by the atomic and molecular environment of the proton spins, such as type, size, and motion of the particles. Consequently, different tissue types or pathological tissue changes characteristically influence *T*_1_ and *T*_2_ times. In CMR, for example, the *T*_1_/*T*_2_ times of myocardium and native blood at 3T are ~1,550/45 ms (12, 13) and 2,000/250 ms (12, 14), respectively. Together with the proton density, this contributes to the image contrast in MRI.

The above set of equations was first proposed by Felix Bloch to describe the temporal dynamics of $\vec{M}$, and has accordingly been named Bloch equations (15, 16). For the evolution of signal intensities, however, this model is less suitable as it requires solving the individual Bloch equations for all magnetization vectors. Instead, the so-called Extended Phase Graph (EPG) model has been proposed (8, 17–19), where signal dynamics can be expressed efficiently based on a rotation matrix formalism in the Fourier domain (see Sections k-space and View Planning and Image Acquisition).

### Image Acquisition: What Is the Position?

Having established the nuclear origin of the MR signal and how it can be manipulated by RF pulses, the next necessary step for image formation is to spatially localize the signal. This is achieved through spatially varying magnetic fields, the so-called gradients. As described in Equation (2), the precession frequency ω_*L*_ of a spin is a function of the magnetic field. Thus, by making the magnetic field a function of the location, spins at different spatial locations will have different resonance frequencies. Although various gradient forms can be applied, linear gradients have proven to be the most useful and, thus, will be assumed in the following description. While a linear gradient field is turned on, ω_*L*_ becomes a function of the spin position $\vec{r}$ and the field gradient $\vec{G}=\;\nabla \vec{B}$:


(5) $$\begin{array}{l} \omega_{L}\left(\vec{r}\right)=\gamma \vec{G}\cdot \vec{r}. \end{array}$$

This principle can be used both to select imaging slices within the body as well as to encode positions in-plane within the slice. For simplicity, we will further assume that the imaging slice is in the transverse *xy*-plane. Note, however, that arbitrary acquisition angles can be achieved by using a combination of the *x*-, *y*-, and *z*-gradients for the encoding described below.

#### Slice Selection (SS)

In slice selection, an additional spatially varying magnetic field gradient ${\vec{G}}_{z}$ can be applied such that the field strength varies along the *z*-axis. Thus, the Larmor frequencies of spins will vary along this axis too: ω_*L*_ = γ(*B*_0_ + *G*_*z*_*z*). While the additional gradient field is turned on, spins in different *xy*-planes precess with different frequencies, while spins within the same plane all precess with frequency ω_*L*_. If the excitation RF pulse is chosen to have just the right frequency bandwidth, only spins in the corresponding *xy*-plane are excited. Accordingly, a transverse magnetization will only be created in those.

#### In-plane Phase Encoding (PE)

After selecting a two-dimensional (2D) slice, the signal needs to be located within the slice. A phase encoding gradient ${\vec{G}}_{y}$ along the *y*-axis is temporarily applied before the readout. During the presence of ${\vec{G}}_{y}$, spins along the gradient axis precess with different frequencies. After ${\vec{G}}_{y}$ has been turned off, the spins will have accumulated different phases, pointing in different directions, but continue to precess with the same frequency. For one gradient strength, only one phase shift can be achieved. Therefore, multiple PE steps are necessary, which primarily determines the overall scan time. In order to acquire a three-dimensional (3D) volume, a second PE gradient along the slice-selection axis can be applied in the same stepwise manner.

#### In-plane Frequency Encoding (FE)

To account for the remaining spatial direction, a gradient ${\vec{G}}_{x}$ is applied, such that spins along the *x*-axis will precess with linearly increasing frequencies. Upon Fourier transforming the signal, each obtained frequency can thereby be connected to a position/pixel on the selected axis, usually the *x*-axis.

### K-Space

In the presence of linear gradient fields, the MR signal can be conveniently expressed with the so-called k-space formalism. If we consider the precession of ${\vec{M}}_{xy}$ in the transverse plane, it can be described as:


(6) $$\begin{array}{l} M_{xy}\left(t,\vec{r}\right)=e^{-i\omega t}M_{xy,0}\left(\vec{r}\right) \end{array}$$

with precession frequency $\omega =\gamma \;B\left(\vec{r}\right)=\gamma \;\left(B_{0}+\;\vec{G}\left(\vec{r}\right)\cdot \vec{r}\right)$ [Equations (2, 5)]. Given that the acquired signal is the sum of the magnetization of all spins in the imaging volume, it can be described as follows:


(7) $$\begin{array}{l} S\;(t)\propto \;e^{-i\gamma B_{0}t}\int e^{-i\gamma \;\vec{G}\;(\vec{r})\cdot \;\vec{r}\;t}M_{xy,0}(\vec{r})\;\vec{dr}. \end{array}$$

The gradient related frequency contribution can be written in terms of the gradient strengths *G*_*x*_, *G*_*y*_ and *G*_*z*_:


(8) $$\begin{array}{l} \gamma \;\vec{G}\left(\vec{r}\right)\cdot \vec{r}\;t=\gamma \left(G_{x}x+G_{y}y+G_{z}z\right)t\\ =\;k_{x}x+k_{y}y+k_{z}z \end{array}$$

with the spatial frequencies *k*_*x*_, *k*_*y*_, and *k*_*z*_. If motion is considered, $\vec{r}$ (spin position) becomes a function of time $\vec{r}\left(t\right)$. Furthermore, each receiving coil *j*, i.e., each receiving channel, has a specific sensitivity $c_{j}\left(\vec{r}\right)$ signal from different spatial points. Combining these with the previous equation yields


(9) $$\begin{array}{l} S_{j}\left(t\right)\propto e^{-i\gamma B_{0}t}∭e^{-i\left(k_{x}x+k_{y}y+k_{z}z\right)}c_{j}\left(\vec{r}\right)M_{xy,0}\left(\vec{r}\right)\\ dx\;dy\;dz \end{array}$$

Equation (9) shows that the measured signal in time domain and the magnetization in spatial domain are connected via Fourier transformation. As a consequence of this relation, the spatial frequency (*k*_*xy*_) and distance (Δ*k*_*xy*_) of k-space points are associated with image resolution and size (field-of-view, FOV):


(10) $$\begin{array}{l} {\text{FOV}}_{x/y}=\frac{1}{\Delta k_{x/y}}\text{ and }\frac{\Delta x}{y}=\;\frac{1}{k_{x/y}}. \end{array}$$

Image acquisition methods can be distinguished by the proportion of the k-space acquired at once: In so-called single-shot sequences all k-space points are sampled in one acquisition, while in segmented methods the k-space is acquired in subsets during multiple repetitions. The overall scan time is, thus, primarily determined by the number of acquired points in the k-space. In this regard, subsampling techniques offer ways to accelerate image acquisition as described in Section Fast CMR: Speeding up Imaging by Acquiring Less Data.

So far, the MR signal has been treated as a continuous function in both space and time. Actual image acquisition, however, is a discretized process characterized by the data sampling rate and image resolution. Hence, the signal/forward model in Equation (9) can be discretized as:


(11) $$\begin{array}{l} \sigma_{j}=E_{j}\;\vec{\rho }+\vec{\eta }, \end{array}$$

with encoding matrix ***E***_*j*_ for coil *j*, initial transverse magnetization $\vec{\rho }$, and thermal noise $\vec{\eta }$ (20). At time point κ and grid point λ, ***E***_*j*_ is given by $E_{j,\kappa ,\lambda }=c_{j}\left(\vec{r}\right)e^{i\Phi \left({\vec{r}}_{\lambda },t_{\kappa }\right)}$. Neglecting relaxation, the phase factor $\Phi \left({\vec{r}}_{\lambda },t_{\kappa }\right)$ accounts for phase accumulation due to time-varying magnetic fields (see Sections Handling motion and Motion Correction).

### Sequence Building Blocks: Time and Order Are Key

**Figure 1:**
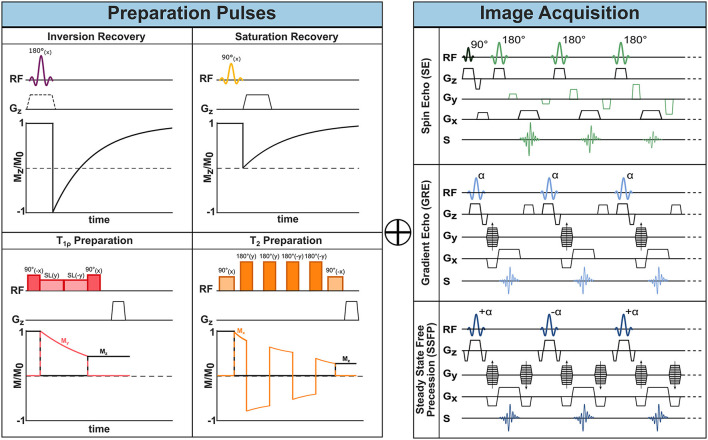
MR sequence building blocks. One or more preparatory pulses (left) can be combined with different acquisition sequences (right) to encode the desired information into the imaging data and achieve different image contrasts.

By manipulating the timing and strength of RF-pulses and gradients, a plethora of MR sequences can be constructed. Different pulse sequences differ in their acquisition speed, encoded image information, or to which degree image contrast is affected by differences in proton density, *T*_1_, or $T_{2}^{*}$, or other properties. CMR sequences are typically described by components for actual image acquisition and components for preparing the magnetization. These elements can be understood as building blocks of MRI sequences. The schematic design of the most common building blocks is shown in Figure 1.

#### Image Acquisition Methods: Get What You Want

##### Spin Echo

As described in the previous section, after RF excitation the net magnetization is subject to $T_{2}^{*}$ relaxation. Fortunately, part of the dephasing of the transverse magnetization can be recovered with a so-called spin-echo (SE) sequence. In this sequence a second RF pulse is applied, where the simplest form comprises a 90° excitation and 180° refocusing pulse. After the first excitation, the spins dephase and fan out in the transverse *xy*-plane. Dephasing caused by temporally invariant field inhomogeneities, however, can be reversed via the second refocusing pulse (21). Its effect is often described as a pancake-flip: The fan of spins is flipped by 180° around the *x*- or *y*-axis, such that the faster spins now move toward instead of away from the slower rotating spins. After a so-called echo time TE, corresponding to twice the time between the two RF pulses, all dephasing caused by static inhomogeneities is rephased and an echo of the signal is created, as depicted in Figure 1. This gives the name to the SE sequence. Consequently, the contrast in SE, is driven by the *T*_2_ time, which captures the residual dephasing caused by temporally variable factors, such as spin-spin interaction.

##### Spoiled Gradient Echo

As opposed to SE, the so-called gradient echo (GRE) sequences retain not the transverse but the longitudinal magnetization. They typically require only one RF excitation pulse after which the frequency encoding gradient is applied (see Figure 1). In GRE, however, the positive FE gradient lobe is preceded by an additional negative lobe.

When the areas of the positive and negative lobe are equal, the initially evoked dephasing of spins is reverted—except for $T_{2}^{*}$ decay. This creates a signal which is referred to as a gradient echo and gives name to the GRE sequence (22). In the so-called spoiled GRE (spGRE), remaining transverse magnetization is destroyed at the end of each TR cycle. This can be achieved with strong gradients at the end of the TR and results in *T*_1_ weighted imaging (23). As no additional RF pulses are required, shorter TE and TRs can be achieved in GRE compared to SE allowing for faster image acquisition. In GRE, the echo signal is subject to $T_{2}^{*}$ decay as no rephasing of field inhomogeneities is achieved. Therefore, GRE sequences are less robust in the presence of field inhomogeneities.

##### Balanced Steady-State Free Precession

A third common image acquisition sequence in CMR is the so-called Balanced Steady-State Free Precession (bSSFP). It can be understood as a hybrid between SE and GRE. Starting from a GRE sequence, a train of RF pulses is applied with very short TR (≪*T*_2_) such that the magnetization never fully recovers between two consecutive RF pulses and a non-zero net magnetization is present at the next RF pulse. This residual magnetization contributes to the signal of the following TR. Characteristically for bSSFP, the flip angles are alternated every TR between +α and −α causing the net magnetization to flip around the *z*-axis between TRs (24, 25). This further means that each RF pulse has both an excitation and refocusing effect on the spins and explains the SE nature of bSSFP sequences. For effective refocusing of the magnetization, the gradient moments on all three axes (SS, FE, PE) need to be zero at each TR. This means that the areas of positive and negative gradient lobes on each axis must be equal, as shown in Figure 1, which is referred to as balanced gradients. The alternating magnetization progresses through a transient state and after a certain number of TR cycles $\vec{M}$ reaches a steady state, that is a stationary amplitude. For TR≪*T*_2_ the contrast in bSSFP sequences is determined by the *T*_2_/*T*_1_ ratio (24). The main advantage of bSSFP lies in the improved signal to noise ratio (SNR) compared with spGRE, due to the recycled transverse magnetization. However, the scheme is highly sensitive to off-resonances making it a less common choice for high field strength and rarely useful for ultra-high fields (25).

### Preparation Pulses: Be Prepared for the Changes

#### Inversion Pulses

So-called inversion pulses, are 180° RF pulses which can be applied before image acquisition in order to flip the initial magnetization along the *B*_0_ axis (26). During the time between inversion and the first imaging RF pulse (inversion time, TI), the longitudinal magnetization recovers along the *B*_0_ axis toward its equilibrium state as depicted in Figure 1. At image acquisition, the degree to which $\vec{M}$ has recovered determines the image contrast and, thus, induces *T*_1_ weighting. This enhances the image contrast based on *T*_1_ properties, which is of interest in many imaging applications. By adjusting TI, imaging can also be timed to the point when the magnetization of specific tissues is crossing the zero point, leading to effective signal suppression (26). For instance, in double inversion black blood imaging (27), a global and slice-selective inversion pulse are applied immediately one after the other such that only the blood outside of the imaging slice is inverted. With an appropriate TI, the signal of blood flowing into the slice can be nulled at image acquisition.

#### Saturation Pulses

Intentionally suppressing tissue signal can also be achieved through so-called saturation pulses. These RF pulses flip the magnetization to the transversal plane. Subsequent spoiler gradients dephase the magnetization, thereby nulling the signal from the “saturated” spins. The subsequent recovery of longitudinal magnetization is shown in Figure 1. Saturation pulses can be made spatially selective, such that regions in or outside of the image are canceled out. For instance, artifacts due to through-slice flow can be reduced by applying a saturation pulse upstream, parallel to the imaging slice. Furthermore, saturation pulses can be made selective to specific chemical species by adjusting the resonance frequency. The most common example is fat saturation, where RF pulses with carrier frequencies specific to ω_*L*_ of fat are applied close to the imaging sequence such that only fat but not water signal is nulled. Creating uniform saturation with common rectangular RF pulses is hindered by their high sensitivity to *B*_0_ and *B*_1_ inhomogeneities. To overcome this limit, adiabatic saturation modules—such as composite (28) or *B*_1_ insensitive rotation (BIR) pulses (29)—have been proposed.

#### T_2_ Preparation

*T*_2_ contrast can be induced using the so-called *T*_2_ preparation pulses (30, 31). In a *T*_2_ preparation, a first 90° excitation pulse is followed by a series of refocusing pulses and, finally, by a 90° flip-back pulse. To induce robust refocusing, the refocusing pulses are separated by a 2τ interval, whereas the interval between the 90° pulses and the refocusing pulses is equal to τ. The total *T*_2_ preparation time is varied to achieve different echo times. During this time, the refocusing pulses compensate for $T_{2}^{*}$-decay, resulting in a transverse magnetization decay effectively characterized by the *T*_2_. The final 90° flip-back pulse brings the remaining transverse magnetization back to the *z*-axis, encoding *T*_2_ contrast in the longitudinal magnetization, which is then imaged during acquisition. Several strategies, such as phase cycling following Malcolm Levitt (MLEV) schemes or using composite pulses, are employed in order to make *T*_2_ preparations more robust to field inhomogeneities (32, 33).

#### T_1ρ_

The relaxation constant in the rotating frame of reference, *T*_1ρ_, is an additional property of tissues, besides *T*_1_ and *T*_2_ times. *T*_1ρ_ contrast can be achieved through spin-lock preparations. A spin-lock module consists of a 90° tip-down pulse followed by a continuous wave RF pulse applied for a certain time τ_*SL*_. During this time the magnetization is locked on the spin-lock axis, and it relaxes back to its equilibrium value following an exponential *T*_1ρ_ decay. Finally, a 90° tip-up pulse is applied after the spin-lock to restore longitudinal magnetization. Spin-lock pulses show high susceptibility to field inhomogeneities. Several compensated schemes, as well as adiabatic spin-lock modules, have been proposed to make *T*_1ρ_ preparation more robust to *B*_0_ and $B_{1}^{+}$ variability (34–36).

### Common CMR Sequences: What Are They Made of

The sequence building blocks introduced in the previous sub-sections can be combined to design tailored sequences to assess, for example, cardiac function and viability. These sequences represent powerful tools for the non-invasive characterization of congenital or acquired cardiovascular diseases, including ischemia, valvular diseases and ischemic and non-ischemic cardiomyopathies, as described in Section Clinical Cardiovascular MR: What do we See and Why do we Need it?. Here, we will discuss the physics principles governing the main CMR sequences and introduce some emerging techniques.

#### Cine bSSFP

Cardiac function is commonly assessed using bSSFP sequences in cine mode. The structure of bSSFP sequences, described in Section Sequence Building Blocks: Time and Order are Key, allows very short TR values to be achieved and increasing the number of k-space lines acquired in a single heartbeat. At the same time, bSSFP sequences maintain high intrinsic myocardial/blood contrast (37). These characteristics enable the fast acquisition of a single slice across multiple cardiac phases (typically 10–30 phases, also referred to as *frames*). This allows the reconstruction of movies of the beating heart. To achieve good spatial resolution for every frame, the acquisition of each frame is divided among different cardiac cycles, using the so-called segmented acquisition (see Section Handling Motion). During each heartbeat, in fact, only a limited number of k-space lines (or a *segment*) is acquired for each cardiac phase. Therefore, several heartbeats are necessary to acquire all the k-space segments. The acquired images are then assigned to the corresponding heart phases using retrospective gating (see Section Handling Motion). Full heart coverage is achieved by repeating the acquisition of each cine image set for different locations and orientations.

#### Late Gadolinium Enhancement CMR

Cardiac viability studies traditionally rely on the use of gadolinium-based contrast agents (see Section Ischemic Heart Disease). These cause enhancement of tissue contrast, with respect to native *T*_1_ contrast. Gadolinium-based contrast agents have the effect of shortening the *T*_1_ of both healthy and diseased myocardium, resulting in their enhancement right after injection. However, healthy and diseased tissues are characterized by different contrast wash-out times: at a certain time point after injection, gadolinium has largely washed out of healthy tissues but is still retained in pathological areas where the extracellular space is expanded.

Late gadolinium enhancement (LGE) imaging is most commonly performed with an inversion-prepared segmented GRE sequence, where the inversion time (TI) is chosen so as to null the signal from healthy myocardium and maximize the contrast. This technique, however, shows high sensitivity to a correctly chosen TI, which is often based on a quick scout acquisition (38). Alternatively, Phase-Sensitive Inversion-Recovery (PSIR) sequences can be used to mitigate the effects of an incorrect TI on the resulting image contrast (39). Unlike traditional IR sequences, PSIR retains the information on the longitudinal magnetization polarity by incorporating the signal phase in the image reconstruction. The reconstructed PSIR images exhibit enhanced contrast between healthy and diseased myocardium. PSIR sequences, however, require the acquisition of a reference image, in addition to the inversion-recovery image, to extract the signal polarity. Nevertheless, the total scan time can be kept constant by acquiring the reference scans during the T_1_-recovery heartbeats.

#### First Pass Perfusion CMR

First pass perfusion CMR is becoming essential for measuring myocardial blood flow (MBF) and detecting myocardial ischemia (40), as described in Section Ischemic Heart Disease. In this technique, images are acquired during the first passing of a bolus of contrast agent, which increases the blood signal as described above. To this end, saturation prepared single-shot GRE (1.5T/3T) or bSSFP (1.5T) sequences in multiple slices are usually performed. In consequence, myocardial regions with low perfusion and, hence, low gadolinium concentration, will exhibit lower signal intensities. Moreover, if perfusion data is acquired under stress conditions, myocardial perfusion reserve can be obtained as the ratio of MBF at stress and at rest. Recent first pass perfusion methods can even yield quantitative MBF values by taking the temporal dynamics of the signal into account (41). In clinical practice, first pass and LGE images are often evaluated alongside each other. This provides additional information on cardiac viability.

### Quantitative CMR Techniques

The methods described in the previous section offer powerful tools for the qualitative assessment of cardiac function and viability. Nevertheless, new quantitative MRI biomarkers have recently been introduced, significantly enhancing the diagnostic capabilities of CMR. Here, we provide a general overview of these techniques.

#### T_1_ Mapping

**Figure 2:**
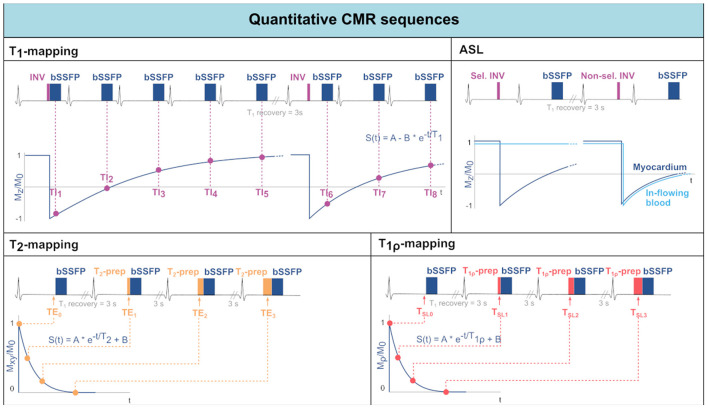
Acquisition schemes for quantitative CMR techniques: *T*_1_-mapping, Arterial Spin Labeling (ASL), *T*_2_-mapping, and *T*_1ρ_-mapping. For each technique, the sequence scheme is represented along with the data sampling and reconstruction strategies.

While *T*_1_-weighted LGE images provide good qualitative characterization of focal myocardial infarction, it becomes less sensitive in the presence of diffuse fibrosis. An emerging alternative is the pixel-by-pixel quantification of *T*_1_ relaxation times (42). By obtaining a healthy reference range, several pathologies can be characterized without the need for healthy reference areas within the image. *T*_1_-mapping can be performed with or without contrast injection. In the latter case, it is referred to as *native* *T*_1_-mapping, as opposed to *post-contrast* *T*_1_-mapping. *T*_1_-mapping sequences are traditionally based on the Look-Locker technique, which consists in measuring the signal at multiple time points following an inversion preparation pulse (43) (see Figure 2). The collected data points, sampling the longitudinal magnetization recovery, are then fit to an exponential curve to derive the *T*_1_ estimates for each pixel. The most commonly used method for myocardial *T*_1_-mapping is the Modified Look Locker Inversion recovery (MOLLI) sequence. Single-shot bSSFP images are each acquired in the end-diastole phase of consecutive heart beats following the application of an inversion pulse (44). A typical MOLLI pattern is the 5(3s)3 scheme (45), where the first inversion preparation is followed by 5 bSSFP acquisitions in separate heart beats, then 3 s of rest are inserted to allow for *T*_1_ recovery and, finally, a second inversion pulse is followed by the last 3 bSSFP measurements. MOLLI enables precise *T*_1_-mapping in a single breath-hold.

Saturation recovery has been proposed as an alternative to inversion-recovery techniques. The SAturation recovery single-SHot Acquisition (SASHA) sequence (46) acquires nine consecutive saturation-prepared single-shot bSSFP images, with variable saturation recovery times, in consecutive hearth beats. Saturation recovery-based sequences have the advantages of not requiring rest periods and of acquiring each image independently. As a result, the *T*_1_-mapping will be less susceptible to biases introduced by *T*_2_, magnetization transfer, inversion pulse efficiency and magnetic field inhomogeneities, however at the expense of a reduced dynamic range and, thus, reduced precision. Hybrid inversion, saturation recovery sequences have also been proposed to mitigate some loss in precision (47).

#### T_2_/${\text{T}}_{2}^{*}$ Mapping

*T*_2_ relaxation time in the myocardium can be used as a marker for the presence of edema, as mentioned in Section Myocardial Inflammation. *T*_2_-mapping is most often performed using a *T*_2_-prepared bSSFP sequence (32), as shown in Figure 2. Commonly, the acquisition of each image is interleaved with rest periods to allow for *T*_1_ recovery. Alternatively, *T*_1_ recovery periods can be omitted introducing a saturation pulse at the end of the R wave in every heartbeat (48). The signal is sampled at different TEs by varying the echo time of the *T*_2_-preparation. Acquired data are then fit to an exponential decay curve to estimate *T*_2_ values.

$T_{2}^{*}$-mapping can also be performed and is used for the identification of iron accumulation (33, 49). $T_{2}^{*}$-mapping is commonly achieved with multi-echo GRE sequences, with a number of equally-spaced echo times. The resulting signal is then fit to an exponential decay curve to estimate $T_{2}^{*}$ values.

#### T_1ρ_ Mapping

Myocardial *T*_1ρ_-mapping has been recently introduced as a promising method for assessment of myocardial fibrosis without the need for exogenous contrast agents (50). *T*_1ρ_-mapping is performed through spin-lock-prepared bSSFP sequences acquired for different spin-lock times and interleaved with *T*_1_ recovery periods (Figure 2). The sampled signal is then fit to an exponential decay curve to estimate the relaxation constant *T*_1ρ_. The *in-vivo* applicability of *T*_1ρ_-mapping, however, is hindered by the susceptibility to field inhomogeneities, especially at high field strengths.

#### Cardiac Magnetic Resonance Fingerprinting

Obtaining *T*_1_/*T*_2_ values with the techniques described above requires the acquisition and subsequent fit of multiple high-resolution images to exponential decay models. Unfortunately, high-resolution scans can be impractically long, particularly if multiple parameters need to be estimated. On the other hand, magnetic resonance fingerprinting (MRF) offers the possibility to simultaneously quantify multiple tissue parameters in a single scan (51). By varying sequence parameters such as TR and FA throughout the acquisition of highly undersampled images, information on tissue parameters is encoded in the temporal signal of each pixel. These so-called fingerprints are unique to the underlying tissue parameter configuration and can be compared to previously generated dictionaries to infer the model parameters of interest. The dictionary contains simulated time signals for the chosen sequence parameters for a range of model parameter values. While MRF is well established for studies of the brain, non-static organs such as the heart pose challenges due to high respiratory and cardiac motion (52, 53). Therefore, cardiac MRF is performed in breath-held acquisitions which are ECG triggered to the quiescent, end-diastolic phase of the cardiac cycle (54). More recently, free-breathing cardiac MRF sequences have also been proposed (55). However, since the heart rate varies over time, multiple dictionaries which are simulated with the actual heart rate, are required. To further increase sensitivity to *T*_1_/*T*_2_, inversion or saturation pulses can be added (54). Although clinical validation is still in its early stages due to complex acquisition and reconstruction as well as relatively long breath-holds, cardiac MRF remains a promising technique for fast multi-parametric mapping.

#### Blood Flow

Cardiovascular flow is typically measured through phase contrast methods that are sensitized to through-plane velocities (56). Flow velocity values are obtained by adding bipolar flow-encoding gradients in the slice-selection direction, after the excitation but before read-out. Flow encoding is based on the principle that moving spins, contrary to stationary spins, accumulate a net phase shift proportional to their velocity when subject to bipolar gradients. By toggling the bipolar gradients, the other contributions to the phase shift, such as those cause by field inhomogeneities, can be neutralized and the blood flow velocity can be quantified.

2D-phase contrast imaging only resolves though-plane flow in 2 spatial dimensions. However, more recently, 4D-flow imaging has been proposed, which combines 3D spatial encoding with 3D directional velocity encoding (57, 58). As a result, 4D-flow MRI offers the possibility to visualize the temporal evolution of complex flow patterns in a 3D volume.

#### Arterial Spin Labeling

CMR allows the assessment of myocardial perfusion (40). However, current techniques are based on first pass perfusion imaging which requires the use of contrast agents and, thus, limits the repeatability and clinical applicability. Arterial spin labeling (ASL), on the other hand, relies on endogenous contrast in the form of magnetically labeled blood. The general idea behind ASL is to acquire two images, one with and one without labeled blood. Subsequently, these images are subtracted to obtain the perfusion related signal only. For cardiac applications of ASL, the most commonly used tagging method is Flow-Alternating Inversion Recovery ASL (FAIR-ASL) (59, 60), depicted in Figure 2. In FAIR-ASL, spatially selective and non-selective inversion pulses are applied alternately: The selective pulse serves as a preparation for the control image. During image acquisition after the non-selective pulse, however, in-flowing inverted spins reduce the longitudinal magnetization proportionally to the perfusion rate. During reconstruction, the subtracted images are first normalized to the baseline intensity, i.e., an image without any preparation pulse. This difference is then multiplied with the inversion efficiency, the blood water-tissue partition coefficient, and an exponential factor accounting for *T*_1_-decay to obtain the MBF (61).

### Common CMR Artifacts: Obscured Reality

**Figure 3:**
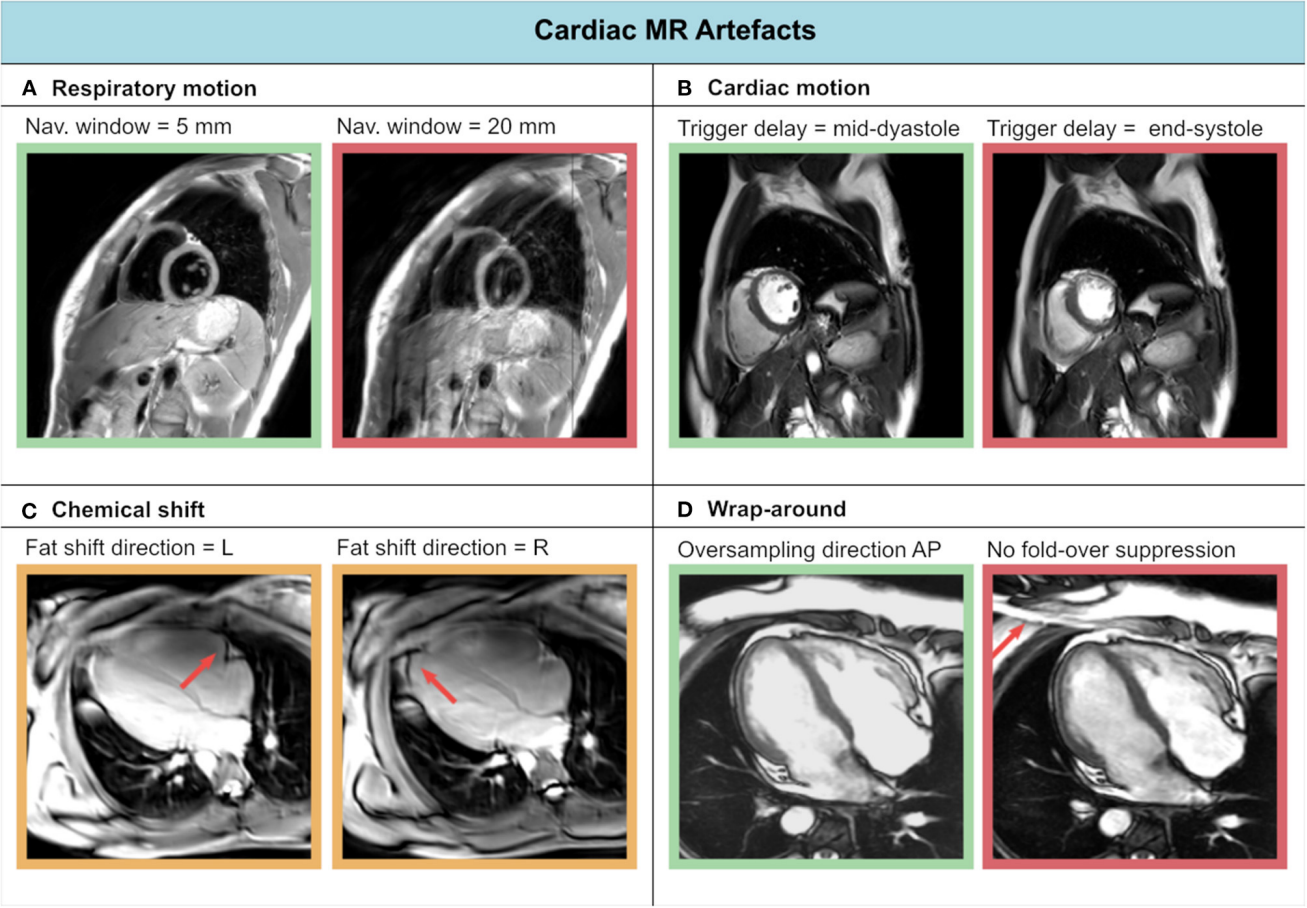
Experienced CMR image artifacts of **(A)** respiratory motion, **(B)** cardiac motion, **(C)** chemical shift, and **(D)** wrap-around.

The complexity of cardiac anatomy, as well as the presence of respiratory motion, cardiac motion, and blood flow, constitute a unique set of challenges for CMR examinations. In this section we recount the most common artifacts in cardiac MR (Figure 3) and strategies for mitigating them (see Section CMR Image Quality: No Free Lunch). A comprehensive guide to cardiac MR artifacts can be found in Ferreira et al. (62).

Respiratory motion can cause inconsistencies between different segments of the acquisition. As a result, ghosting artifacts may appear on the reconstructed images. Two approaches are commonly used to avoid breathing-related artifacts: breath-holding and respiratory navigators (both will be described in Section Handling Motion). On the other hand, cardiac motion can cause blurring for long imaging blocks, when the acquisition window includes phases of rapid motion. This effect is commonly tackled by introducing cardiac triggering, which synchronizes the acquisition with the cardiac cycles. Choosing relatively long trigger delays from the R peak of the electrocardiogram (ECG) signal enables acquisition during quiescent cardiac phases, such as mid-diastole.

Blood flow can also be a cause of artifacts in CMR. As already discussed in the blood flow imaging paragraph of Section Quantitative CMR Techniques, motion-induced phase shifts occur in presence of blood flow, corrupting the spatial phase encoding. Flow-compensated gradients can be employed to minimize these alterations, by nulling the higher-order gradient moments. For instance, 1st order flow compensation consists of nulling the gradients' 1st order moment, minimizing constant flow velocities contributions.

Aliasing artifacts are very common in MRI and specifically in CMR, where the strict time constraints often limit the FOV dimensions. These artifacts manifest as wrap-around ghosts, which can overlap to the anatomical structures under investigation. While aliasing in the frequency-encoding direction can be avoided through oversampling, this is not feasible in the phase encoding direction without increasing scan time. In this case, the FOV must be enlarged at the expense of lower resolution.

Finally, chemical shift artifacts can manifest in the presence of pericardial fat. These arise because of the different molecular environment of protons in fat and water, whose resonant frequencies differ by approximately 420 Hz (at 3T) as a result. This difference results in a misregistration of fat and water tissues along the frequency encoding direction. Chemical shift artifacts become more evident, for example, when changing the frequency encoding direction. They can be reduced by increasing the signal bandwidth, albeit at the cost of lower SNR.

## Clinical Cardiovascular MR: What Do We See and Why Do We Need It?

This section will outline the contributions CMR can make within each of the major cardiovascular subspecialties and set the scene for the remaining sections in this manuscript which focus on image acquisition, reconstruction, and the burgeoning impact of artificial intelligence on all these areas. Where relevant, reference is made to international diagnosis and treatment guidelines and the levels of supporting evidence underpinning recommendations.

### Basic Principles and Advantages of CMR: What You See and What You Get

CMR is widely recognized as the gold-standard for the non-invasive quantification of left ventricular (LV) ejection fraction which remains a cornerstone parameter that guides decision making in various scenarios ranging from the diagnosis of heart failure to determining the need for primary prevention implantable cardioverter defibrillators (ICDs) and the timing of surgical intervention in patients with valvular heart disease (63, 64). For many of these applications, echocardiography remains a first-line investigation, but CMR is particularly valuable for evaluating cardiac structure and function in patients with poor acoustic windows. This is recognized in the recent European society of cardiology (ESC) heart failure guidelines as a class I indication for CMR (Class I: evidence and/or general agreement that a given treatment or procedure is beneficial, useful, or effective) with level of evidence C (consensus opinion of experts and/or small studies, retrospective studies, registries) (63). The ability to non-invasively acquire high spatial and temporal resolution images in any plane using bSSFP sequences which have high intrinsic T_1_ and T_2_ contrast affords high endocardial definition enabling chamber volumes and function to be quantified with high accuracy and precision (65). This is achieved by acquiring a contiguous short axis stack parallel to the atrioventricular groove and planned with two and four chamber cine sequences (5, 66), see Section Plan Imaging Accurately and Avoid Common Mistakes.

A key feature of CMR is its ability to non-invasively characterize tissue by exploiting intrinsic differences in nuclear magnetic relaxation characteristics of hydrogen nuclei which are found in abundance in the human body in different chemical environments in the form of water but also bound in large macromolecules such as triglycerides and proteins (Supplementary Figure 1). This enables different anatomical structures and pathology to be readily appreciated and differentiated without the need for exogenous contrast. However, the administration of the latter, in the form of large macromolecular chelates of the paramagnetic element gadolinium, augments our ability to detect pathology even further by highlighting the presence of myocardial fibrosis, infiltration, or areas of infarction (67). Gadolinium contrast agents shorten T_1_ relaxation times in proportion to their local concentration. As large positively charged macromolecules, they are unable to penetrate the intact cell membrane and so remain entirely extracellular. As such, in tissues where the extracellular space has been expanded by the presence of fibrosis or infiltrated by exogenous proteins such as for instance in cardiac amyloidosis, gadolinium can accumulate to higher local concentrations. If imaged ~10 min after contrast administration using an appropriate inversion recovery prepared T_1_-weighted sequence with an inversion time set to null the signal from healthy myocardium, such areas are illuminated as gadolinium washes out of healthy tissue but remains at higher concentrations in diseased areas, causing faster recovery of signal. Infarcted or non-viable areas of myocardium can be similarly delineated as they are rich in extracellular matrix and proteins, but cell-poor or in the case of acute myocardial injury, may be populated by necrotic cells with disrupted cell membranes (68). The LGE imaging technique (see Section Common CMR Sequences: What are They Made of) plays a pivotal role in phenotyping patients with heart failure, particularly differentiating patients with ischemic from non-ischemic heart failure (Class IIa: conflicting evidence and/or divergence of opinion about the usefulness/efficacy of the given treatment or procedure but weight of evidence/opinion is in favor of usefulness or efficacy) with level of evidence C (63).

### Ischemic Heart Disease

**Figure 4:**
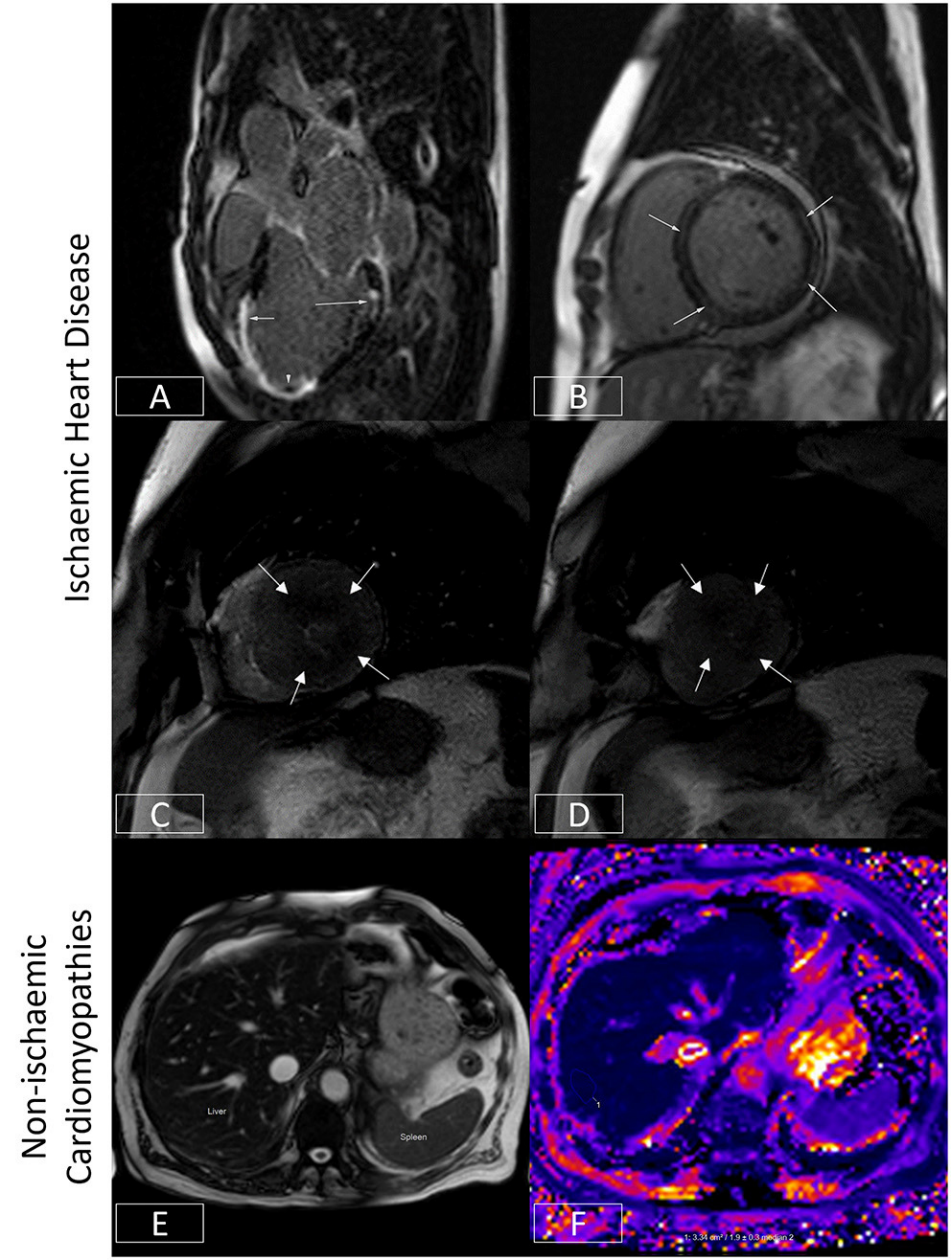
Ischaemic and non-ischaemic heart disease. **(A)** Late gadolinium enhancement sequence in the 3-chamber view. There is near transmural sub-endocardial enhancement of the mid-apical septum and apex (short arrow, mid-left anterior descending coronary artery territory). A signal void focus is also seen adherent to the apex (arrowhead). This represents a left ventricular thrombus. In addition, there is focal partial thickness sub-endocardial enhancement of basal inferolateral wall (long arrow, circumflex coronary artery territory), which spares the sub-epicardium (denoting an ischaemic etiology). The presence of infarcts in two different coronary territories alludes to the potential presence of multivessel coronary disease. **(B)** Late gadolinium enhancement sequence demonstrating a ring or circumferential pattern of non-ischaemic enhancement. The areas of enhancement involve the mid-wall or sub-epicardium, sparing the sub-endocardium. **(C,D)** Stress perfusion scan from a patient with hypertrophic cardiomyopathy. There is widespread circumferential sub-endocardial delayed arrival of contrast (hypoperfusion) at mid-ventricular level **(C)** and apex **(D)**, typical of microvascular dysfunction. **(E,F)** Bright blood axis scout at upper abdominal level **(E)**. The normal liver should have signal characteristics similar to the spleen (marked). However, in this patient with hepatic iron overload, the spleen appears almost black due to accelerated dephasing of spins brought about by the increasing field inhomogeneity generated by intrahepatic iron stores. This ${\text{T}}_{2}^{*}$ effect can be used to quantify liver iron levels **(F)**. Here, the liver ${\text{T}}_{2}^{*}$ is ~1.9 ms, denoting moderate hepatic iron overload (normal > 6.3ms) equivalent to ~5–10mg iron/g dry weight.

In patients with ischemic heart disease, occlusion of an epicardial coronary artery tends to cause injury and necrosis of endocardial cells first as these are furthest away from the blood supply, evolving to a wavefront of necrosis that gradually spreads centrifugally toward the epicardium (Figure 4A). Areas of LGE extending from the sub-endocardium, particularly if they are regional or in a coronary distribution, denote areas of ischemic infarction. In contrast, non-ischemic pathologies such as dilated cardiomyopathy or myocarditis tend to be associated with LGE in an epicardial or mid-wall distribution, allowing ischemic and non-ischemic etiologies of heart failure to be readily distinguished (Figure 4B). CMR is regarded as a class I indication for evaluating acute chest pain or myocardial injury in patients with unobstructed coronary arteries (level of evidence B: moderate quality evidence from one or more well-designed, well-executed non-randomized studies, observational studies or registry studies or meta-analyses of such studies) (69). As well as being diagnostically valuable, it is increasingly being recognized that the presence and/or extent or pattern of LGE may have prognostic significance (70–74).

In patients with ischemic heart disease, the distribution of LGE can localize infarcts to specific coronary territories (Figure 4A), and the transmural extent can determine the likelihood of underlying myocardial viability (75). By imaging the first pass of contrast through the myocardium under conditions of vasodilator stress (typically achieved with adenosine or regadenoson), myocardial perfusion abnormalities may be identified which may signify myocardial ischemia (76). When the epicardial coronary arteries are unobstructed, contrast arrives synchronously and homogeneously in all supplied myocardial segments. However, where there is a hemodynamically significant stenosis in a given coronary artery, that vessel will already be maximally vasodilated at baseline. The administration of a vasodilator will thereby augment blood flow (and so the arrival of contrast) to unobstructed coronary arteries, allowing areas of hypoperfusion to be delineated by the delayed and reduced arrival of contrast to the already maximally dilated stenosed vessel (76). This technique can therefore be used to diagnose the presence of coronary disease (77) or where this is already known, determine the functional significance of a given stenosis identified using an anatomical imaging technique such as invasive coronary angiography or CT coronary angiography. As mentioned previously, this technique is frequently used in tandem with LGE imaging to assess for myocardial ischemia and viability and thereby determine the need for or to guide revascularization (76). Recent US chest pain guidelines now regard this as a class I indication for stress CMR (level of evidence B) (69). Advances in sequence design, image processing, and quantification techniques now enable myocardial blood flow to be measured at the voxel level with high in-plane spatial resolution (78–83). The latter allows microvascular dysfunction to be elucidated non-invasively (79, 84, 85) (Figures 4C,D), and for ischemic burden to be accurately calculated (81, 86). Quantification techniques also appear to improve the ability to correctly identify multivessel coronary disease (87).

### Non-ischemic Cardiomyopathies

The ability to quantify tissue characteristics has enabled various MR relaxation parameters to be used as biomarkers for diagnosis and to guide therapy (42, 88). The seminal example of this is the development of ${\text{T}}_{2}^{*}$ imaging (Figures 4E,F), which has enabled non-invasive hepatic and myocardial iron quantification (49). By allowing the early diagnosis of iron overload cardiomyopathy and timely initiation and titration of chelation therapy, this has been credited with significantly reducing the risk of death from heart failure in patients with thalassemia (89). The development of T_1_ mapping techniques (see Section Quantitative CMR Techniques) has found applications in detecting interstitial fibrosis, and by measuring post-contrast T_1_ together with the knowledge of the patient's hematocrit, the estimation of extracellular volume fraction (ECV) has made it possible to track pathologies such as cardiac amyloidosis (42). This is of growing relevance as these conditions are increasingly amenable to novel therapeutics which can stabilize or even potentially partially reverse cardiac amyloid deposition (90). Thus, CMR is regarded as a class I indication for the evaluation of infiltrative disease and suspected iron overload (level of evidence C) (63).

CMR also plays a vital role in the evaluation of patients with heart failure or suspected non-ischemic heart muscle disease. It can be used as a gatekeeper for invasive coronary evaluation (91) but also to accurately evaluate areas of the heart that are difficult to clearly visualize by echocardiography such the LV apex or the right ventricle. This can be invaluable for the diagnosis of particularly the apical variants of hypertrophic cardiomyopathy (92) and arrhythmogenic right ventricular (RV) cardiomyopathy (93).

### Myocardial Inflammation

**Figure 5:**
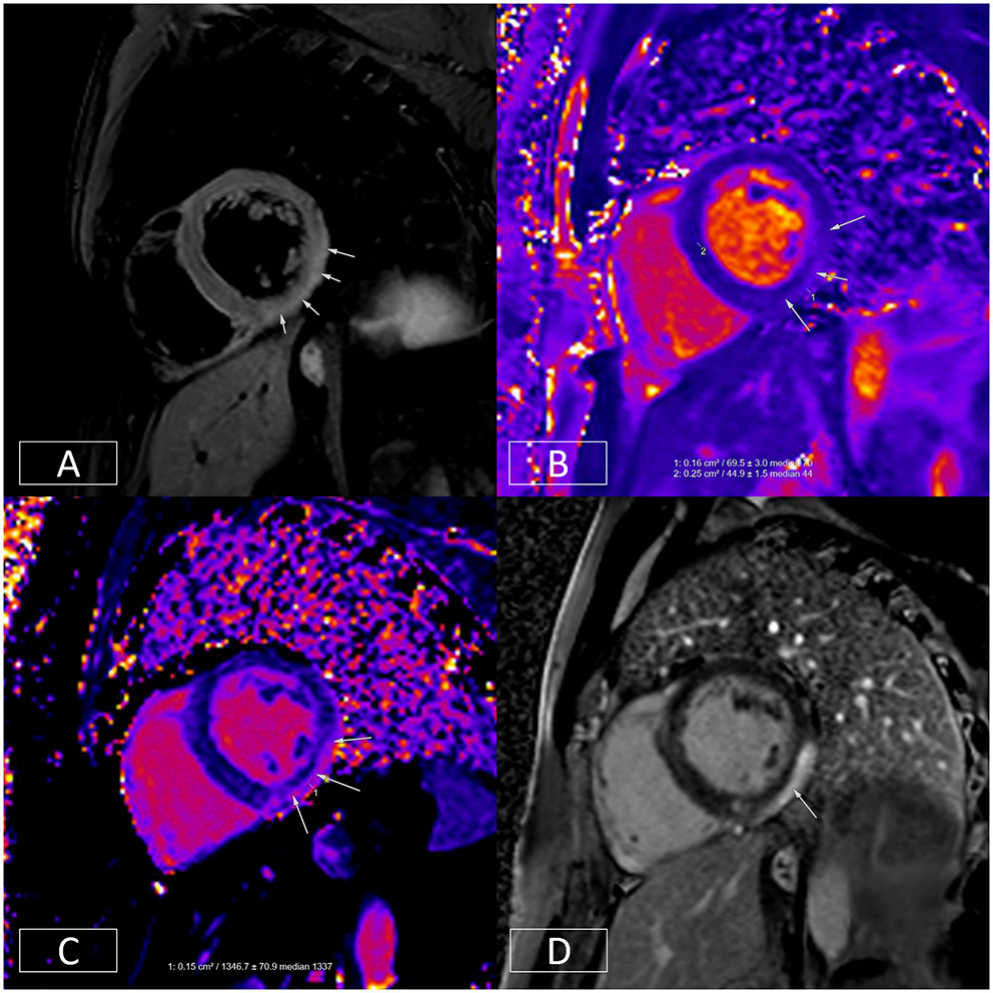
Multiparametric evaluation of a patient with acute myocarditis. **(A)** Depicts increased T_2_ signal in the mid-inferior and lateral walls in an epicardial to mid-wall distribution. The absolute T_2_ time in the inflamed area is increased to ~70 ms **(B)** whereas the remote myocardium in the septum has a normal T_2_ time of 45 ms (normal < 55 ms). **(C)** depicts increased native T_1_, another marker of tissue injury. This is raised at 1,347 ms in the epicardium of the mid-inferior and lateral walls (normal range: 890–1,035 ms on this platform at 1.5T). **(D)** illustrates epicardial to mid-wall enhancement of the mid-inferior and lateral walls, which spares the sub-endocardium (typical of myocarditis).

The ESC guidelines regard CMR as a class I indication (level of evidence C) for the evaluation of patients with suspected myocardial inflammation (63). Acute inflammatory processes and tissue injury can increase tissue water content and increase the mobility of tissue water protons (94). This can be exploited with T_2_-weighted imaging techniques and quantitative mapping methodologies (see Section Quantitative CMR Techniques) to diagnose the presence and distribution of myocardial inflammation (Figure 5) (88, 94, 95). Myocarditis can be diagnosed when in the appropriate clinical context, there is evidence of tissue oedema and inflammation/injury on one T_2_-based (T_2_-weighted-imaging or T_2_-maps) and one T_1_-based criterion (native T_1_ map, LGE imaging, or ECV maps), respectively, in a non-ischemic distribution (96).

### Cardiac Electrophysiology

**Figure 6:**
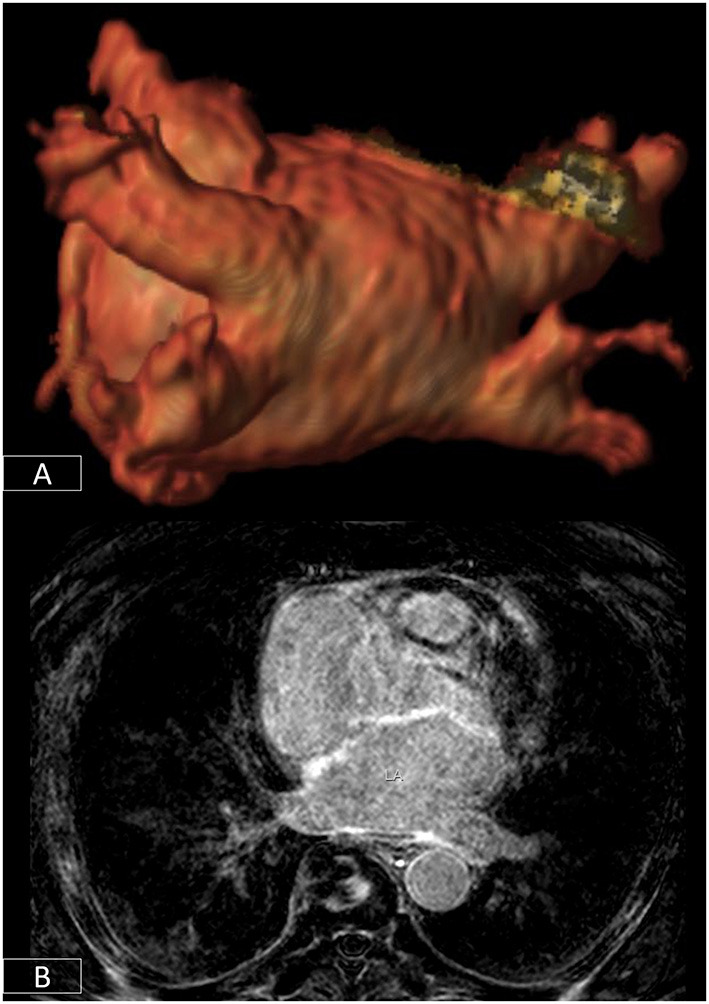
3D-segmentation of the left atrium depicting left atrial anatomy and four pulmonary veins and their tributaries **(A)**. There is extensive fibrosis of the left atrial wall **(B)** on 3D late enhancement sequences which may reduce the likelihood of successful ablation.

Within the sphere of cardiac electrophysiology, not only is CMR playing a vital role in the identification of patients at increased risk of arrhythmia (70, 72–74), but it is increasingly being used to plan invasive arrhythmia ablation procedures (97). Atrial fibrillation is the commonest sustained cardiac arrhythmia and an important cause of morbidity and mortality (98). In most patients, the arrhythmia is triggered by electrical activity from the pulmonary veins which can be treated by electrically isolating these through ablation (98). 3D-anatomical and fibrosis imaging sequences can help to define the number of pulmonary veins and the degree of fibrotic remodeling of the atrium which may influence procedural success (Figure 6) (99). For patients with malignant ventricular arrhythmias, identifying the precise origin of arrhythmic foci often requires prolonged and tedious pace-mapping of the electrical substrate increasing procedure times and thereby risk to patients (100). This can be considerably facilitated by pre-procedural CMR which can identify areas of scar tissue and help target electrical interrogation of the diseased myocardium (100).

### Congenital Heart Disease

CMR has also revolutionized the care of patients with congenital heart disease, which occurs at a frequency of 6–8 per 1,000 live births (101). Advances in care now mean that more patients are surviving to adulthood and so are forming an important cohort of patients who require regular clinical and imaging evaluation (101–103). The complexity of disease can range from minor anomalies such as a small restrictive ventricular septal defect through to patients with complex cyanotic heart disease with cardiac malformations that require often multiple complex surgeries to correct or palliate. The imaging assessment of such patients requires the ability to image in multiple planes, in 3D, and to quantify blood flow, particularly to diagnose the presence and severity of any intracardiac shunts (101, 103). Importantly, this is achieved without the need for any ionizing radiation (which would have a greater impact on this younger cohort of patients who need frequent serial imaging) and unfettered by limitations imposed by acoustic windows as echocardiography often is. This is particularly true for structures such as the right ventricle that are more challenging to image with echocardiography (104). The high accuracy and precision of the measurements of ventricular size and function as well as blood flow enable these parameters to be used to guide the timing for surgical intervention, for instance, pulmonary valve interventions in patients with repaired tetralogy of Fallot (103, 105). The broad utility of CMR in congenital heart disease has been recognized in recent international guidelines (106). The presence of RV scar detected by LGE-CMR has been highlighted as a risk factor for sudden cardiac death and its use for risk stratification is recommended as a class IIa indication (level of evidence C). These guidelines also recognize CMR with physical stress as a class I indication (level of evidence C) for the evaluation patients with coronary anomalies to confirm/exclude myocardial ischemia (106).

### Valvular Heart Disease

While Doppler echocardiography is rightly considered the modality of choice for the evaluation of patients with valvular heart disease, phase-contrast velocity mapping is particularly adept at quantifying regurgitant lesions such as aortic and pulmonary regurgitation (107). It can play a role in corroborating echo findings or in providing accurate quantification where unfavorable echo windows preclude this, or jet eccentricity can result in underestimation of jet severity (Supplementary Figures 2, 3) (64, 108). As in many other spheres of cardiovascular medicine, an accurate quantification of ventricular ejection fraction may be vital in determining the timing of any intervention (109).

### Angiography and Vascular Disease

**Figure 7:**
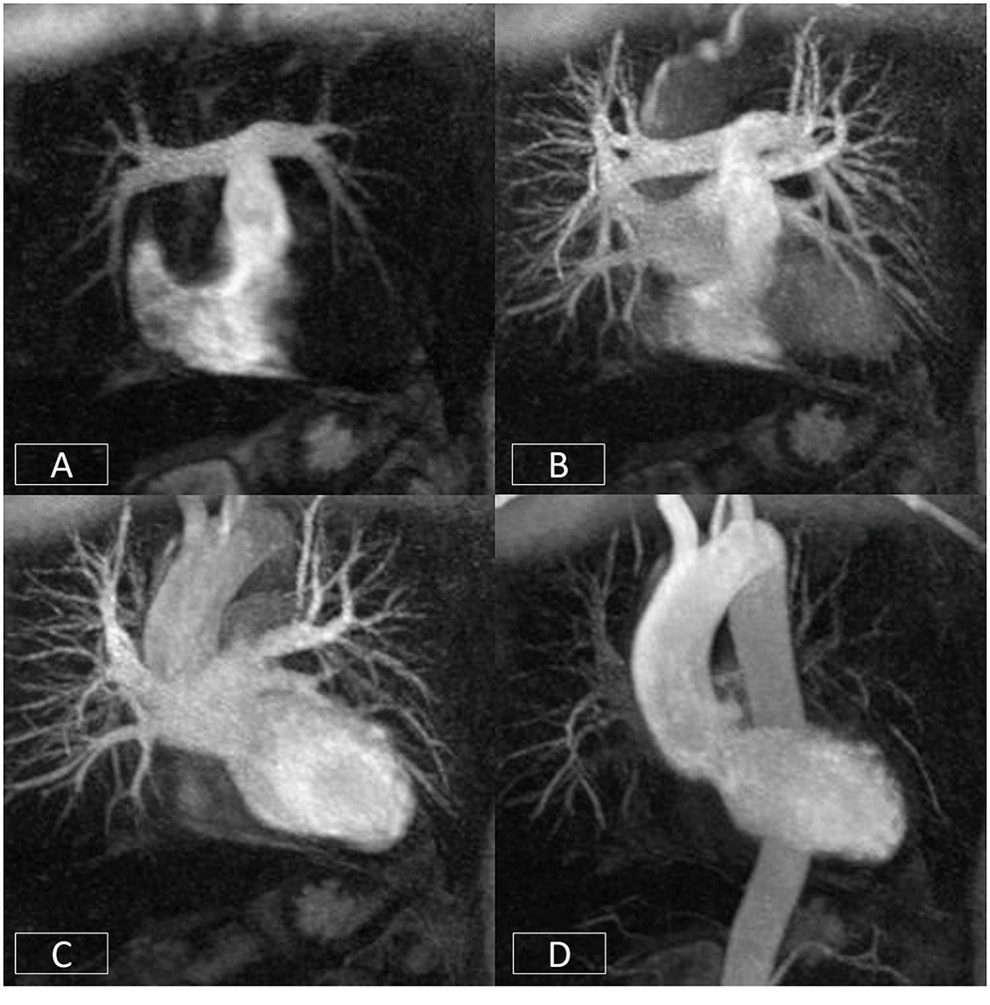
Cardiovascular time resolved 3D-angiography. The bolus of contrast is imaged progressively as it passes from the right side of the heart **(A)** into the pulmonary arteries **(B)**, left atrium/ventricle **(C)**, and thoracic aorta **(D)**. This obviates the need to precisely time the contrast volume and enables the rapid visualization of different parts of the circulation with a single bolus of contrast.

CMR also has the added advantage of enabling visualization of the aorta and great vessels which can often need intervention in patients with aortic valve disease, particularly if this is associated with aortopathy such as patients with bicuspid aortic valves. This can be achieved using time-resolved angiographic approaches (110), as well as with 3D-sequences acquired in free-breathing that can increasingly be combined with multiple tissue contrasts (111–113). The former can enable the visualization of multiple vascular beds and structures (systemic venous, pulmonary arterial and venous, and systemic arterial) with a single dose of contrast (Figure 7) (110). This has a range of applications from the evaluation of vascular disease itself to planning interventions.

Advances in rapid imaging techniques, catheter technology, and the development of interventional imaging suites now allows actual invasive procedures to be performed under MR-guidance (114, 115). This brings the principal benefit of minimizing the need for X-ray fluoroscopy particularly in younger patients who require frequent serial evaluation.

There is also growing interest in leveraging the tissue characterization capabilities of CMR to evaluate coronary plaque characteristics (116, 117). Specifically, T_1_-weighted non-contrast coronary imaging can be used to delineate the presence of methemoglobin, a marker of coronary thrombosis or intraplaque hemorrhage, which has been associated with vulnerable plaque morphology and angina severity (118).

### Cardiac Tumors

Another area where CMR has made significant indispensable contributions to patient care is the evaluation of cardiac tumors (119). While these are thankfully rare, the ability of CMR to provide full-spectrum non-invasive characterization can help to refine the diagnosis and, in many instances, can type specific lesions. Anatomical and cine sequences can localize a lesion and define its geometry and relationship with surrounding structures (119). Sequences with different T_1_ and T_2_ weighting with and without fat-saturation can be used to delineate tissue characteristics. Imaging of the tumor during the first pass of contrast can depict its vascularity and perfusion (120). Imaging in the early phase after contrast administration can differentiate thrombus from neoplasia or reveal the presence of superadded thrombosis. Imaging in the late phase can provide information on the contrast uptake characteristics of the lesion which again can be valuable in differential diagnosis (119, 120). Such data can increasingly be combined with fluorodeoxyglucose (FDG)-positron emission tomography (PET) and other radiotracer uptake data in hybrid CMR-PET imaging platforms to provide truly multimodal comprehensive evaluation that encompasses tumor metabolic activity (121).

In summary, CMR has found applications within every sphere of cardiovascular medicine and has often had a positive disruptive effect—improving diagnosis and in many cases, changing patient outcomes. In a single comprehensive study, it is now possible to assess and reliably quantify cardiovascular anatomy, function, tissue T_1_, T_2_, ${\text{T}}_{2}^{*}$, ECV, perfusion at stress and rest, late gadolinium enhancement, and blood flow. While many of the necessary sequences are ECG-gated and have been done with breath holding, recent advances now make it possible to acquire most data using free-breathing techniques making CMR more accessible and tolerable for patients with cardiovascular disease who often suffer from breathlessness (see Section Handling Motion). However, although the ability to acquire more and more data has grown over the years, the time available to scan patients (typically 1 h) and report the voluminous imaging data sets that are generated has not. This requires careful protocolling and efficient image acquisition to harness the true benefits of this technology in a value-conscious and efficient way (see Section Clinical Cardiovascular MR: How Should we Perform the Examination). Advances in the application of artificial intelligence to both image reconstruction and interpretation may help offset some of these challenges and are addressed in Section Artificial Intelligence for Cardiovascular MR.

## Clinical Cardiovascular MR: How Should We Perform the Examination

As new imaging techniques are developed and the clinical applications of CMR expand, implementing efficient workflow practices has become increasingly important in clinical practice. To complete a comprehensive examination in a clinically acceptable timeframe with high quality imaging requires considerable forethought and planning.

Developing and applying a systematic approach to all aspects of the examination can save considerable scanner time, even if the operator is proficient in the placement of imaging planes. In this section, key areas essential to developing an efficient and structured approach to a CMR examination are outlined.

### Clinically-Tailored Protocols: Make It Right for Patients

The vast array of CMR imaging sequences now available has the potential to considerably extend the CMR examination to clinically unrealistic lengths. Therefore, it is important to approach CMR as a modality with a suite of standardized, clinically-targeted protocols rather than a single one-size-fits-all examination. Protocols should be developed to answer the clinical question with a focus on adding value. Resources are available (5) to guide the development of in-house clinical protocols, which can then be modified to suit patient-specific requirements. It is essential to review each patient's clinical history and previous imaging and tailor the protocol to answer the clinical question, focusing on providing the information only CMR can provide. Even reasonably fit patients can become fatigued from multiple breath holds. Removing any sequences from the examination that do not assist in making the diagnosis will increase efficiency and improve patient compliance.

### Template Protocols: Have Them Ready

Before creating comprehensive CMR protocols on the scanner, build a high quality clinically-appropriate template protocol for each of the basic pulse sequence types, e.g., cine bSSFP; phase contrast (PC) flow quantification imaging; dark blood T_2_ weighted fast spin echo imaging; and LGE imaging (see Sections Preparation Pulses: Be Prepared for the Changes and Common CMR Sequences: What Are They Made of).

In accordance with field strength and scanner capabilities, each template protocol should be created ensuring the scan times are as short as possible whilst maintaining appropriate spatial and temporal resolution, and without introducing artifacts from undersampling or cutting corners (see Section Common CMR Artifacts: Obscured Reality).

Once created, each pulse sequence template protocol can be used and modified to build plane-specific image acquisitions, for example, the 4-chamber or LV vertical long axis views.

This approach ensures consistency and standardization of image quality across the entire examination and clinical service.

### The Building Blocks of a Successful CMR Exam

Almost all CMR examinations will require the basic building blocks of LV and often RV function. All the basic cardiac planes are aligned relative to the heart and are specific to the patient's anatomy. Each plane is prescribed building on prior knowledge from previous acquisitions. Scanning efficiency can be significantly improved by giving careful thought to the order of acquisition of these basic building block sequences. The sequence order below has been planned to ensure that there is no downtime between acquisitions. All image planes required for planning have been acquired at least one acquisition ahead.

### Imaging Protocol for LV and RV Function

1. Three plane (axial, sagittal, coronal) localizer—centered on heart in three planes.
2. Axial non-cine bSSFP localizer—cover from aortic arch to the inferior border of the heart.
3. LV Vertical Long Axis (VLA) cine bSSFP localizer—use the axial bSSFP localizer to prescribe a single slice through the middle of the mitral valve to the LV apex.
4. Sagittal oblique Main Pulmonary Artery (MPA) cine bSSFP—prescribe one slice through the middle of the MPA and the RV outflow tract (RV OT) using the axial localizer.
5. LV short axis (SAX) single heartbeat multislice localizer—use the axial localizer and LV VLA localizer to prescribe a stack through the atrio-ventricular valve.
6. Coronal oblique MPA cine bSSFP—use Sagittal Oblique MPA to prescribe one slice through middle of MPA, Pulmonary Valve (PV) and RV OT.
7. 4-chamber cine bSSFP—use the basal slice of the LV SAX stack localizer to prescribe one slice through the center of the mitral and tricuspid valves. Cross reference to the LV VLA localizer to ensure the slice is through the center of the mitral valve and the LV apex.
8. LV 2-chamber cine bSSFP—use the 4-chamber to prescribe one slice through the middle of the mitral valve to the LV apex.
9. RV VLA cine bSSFP—use the 4-chamber to prescribe one slice through the middle of tricuspid valve to the RV apex. Cross-reference to LV SAX stack localizer to ensure RV OT and PV are in the plane of the slice.
10. LV SAX cine bSSFP-−8 or 6 mm slice thickness with 2 or 4 mm gap, respectively, to make total 10 mm; use both the 2-chamber and 4-chamber diastolic phase images to prescribe a series of slices from the mitral valve annulus to the LV apex. See Section Plan Imaging Accurately and Avoid Common Mistakes for extra positioning tips.
11. Three chamber cine bSSFP—use the basal slice of the LV SAX series and prescribe one slice through the middle of the aortic valve and the left atrium.
12. LV Outflow Tract (LV OT) cine bSSFP—use the 3-chamber to prescribe one slice through the middle of the aorta and the LV OT.
13. RV SAX cine bSSFP—(8/2 or 6/4 mm); use both the sagittal MPA and the RV VLA diastolic phase images to prescribe a series of slices in a plane perpendicular to a line from the pulmonary valve to the apex of the RV. The first slice should be placed at the level of the PV in diastole (122). See Section Plan imaging Accurately and Avoid Common Mistakes for extra positioning tips.
14. Phase Contrast (PC) Flow Aorta—use both the 3-chamber and LV OT diastolic phase images to prescribe a slice perpendicular to the aorta in both planes, at the level of the sino-tubular junction.
15. PC Flow Aortic Valve—use both 3-chamber and LV OT diastolic phase images to prescribe a slice perpendicular to the aorta in both planes, at the level of the aortic valve annulus.
16. PC Flow MPA—use both sagittal and coronal MPA diastolic phase images to prescribe a slice perpendicular to the MPA in both planes, just distal to the valve, through the tubular portion of the MPA, avoiding bifurcation.

### Plan Imaging Accurately and Avoid Common Mistakes

Due to the variability of cardiac morphology and body shape between patients, it can take considerable time to become proficient at localizing cardiac imaging planes. The heart does not lie in an orthogonal plane to the thorax and therefore more than one localizer plane is necessary for accurate and reproducible positioning. Learning to avoid common positioning errors can improve scanning efficiency and diagnostic quality.

#### The 4-Chamber View

**Figure 8:**
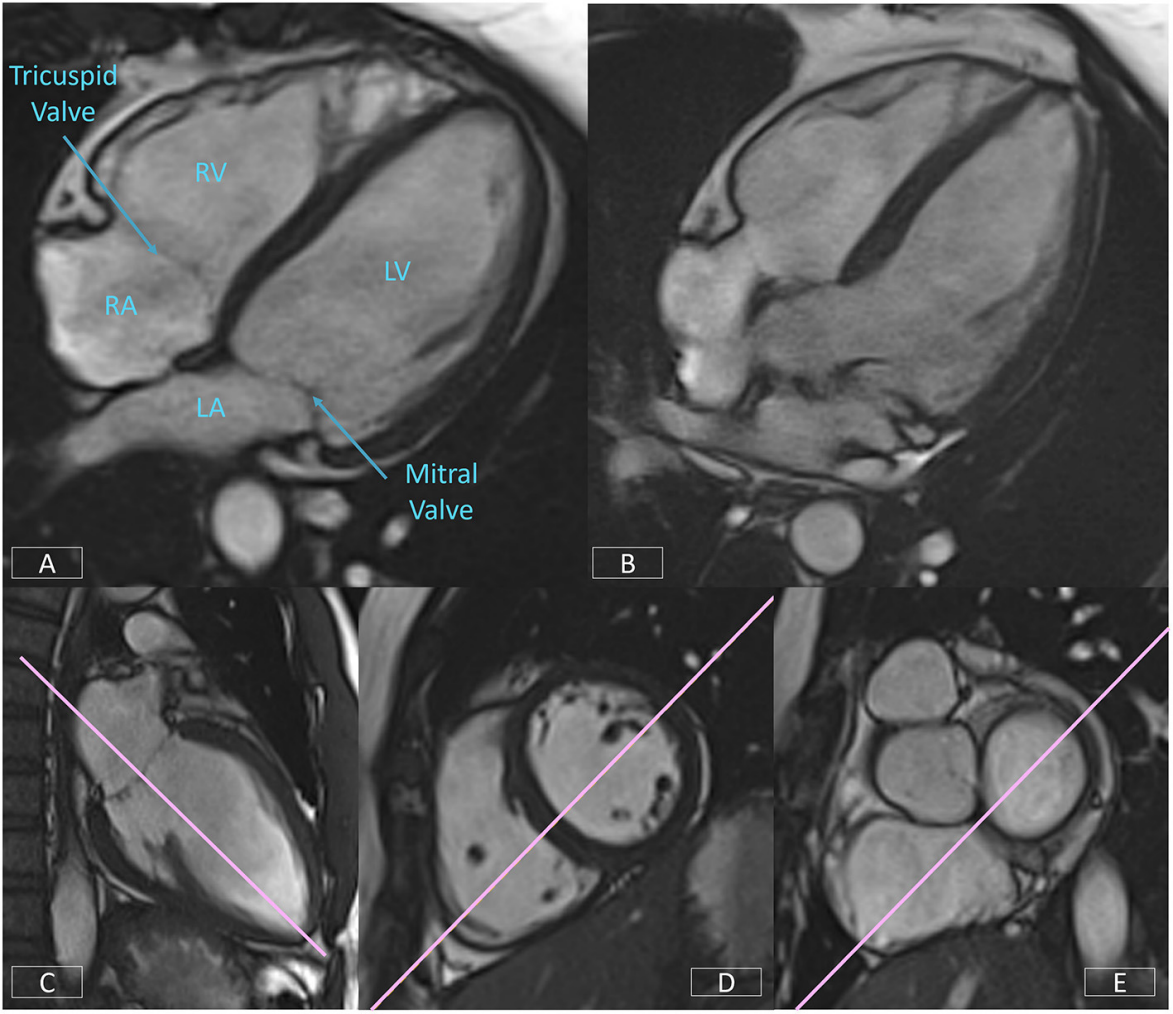
Well-positioned 4-chamber view **(A)** demonstrating mitral and tricuspid valves, right and left atria, and ventricles. Incorrect prescription **(B)** with the slice plane prescribed through the LV OT. Accurate positioning of the 4-chamber view requires the use of three views, the LV VLA view **(C)**, mid-ventricular LV SAX slice **(D)**, and the basal LV SAX slice **(E)**.

The 4-chamber view affords an overall visual assessment of cardiac function. A well-positioned view (Figure 8A) will demonstrate the mitral and tricuspid valves and the right and left atria and ventricles. However, frequently the four cardiac chambers and the atrio-ventricular valve planes are not well-visualized due the slice plane being prescribed incorrectly. Figure 8B is an example of a poorly positioned 4-chamber with the slice plane prescribed through the LV OT. To successfully position the 4-chamber view requires the use of three views. On the LV VLA view (Figure 8C), the operator should ensure the slice plane is prescribed through the center of the mitral valve and the LV apex. On a mid-ventricular LV SAX slice (Figure 8D), the plane is tilted down to the RV apex. Finally, the position is cross-checked on a basal LV SAX view (Figure 8E) to ensure the slice positioning avoids the LV OT and aortic root.

#### Left Ventricular Short Axis—Accurate Positioning of the Basal Slice

**Figure 9:**
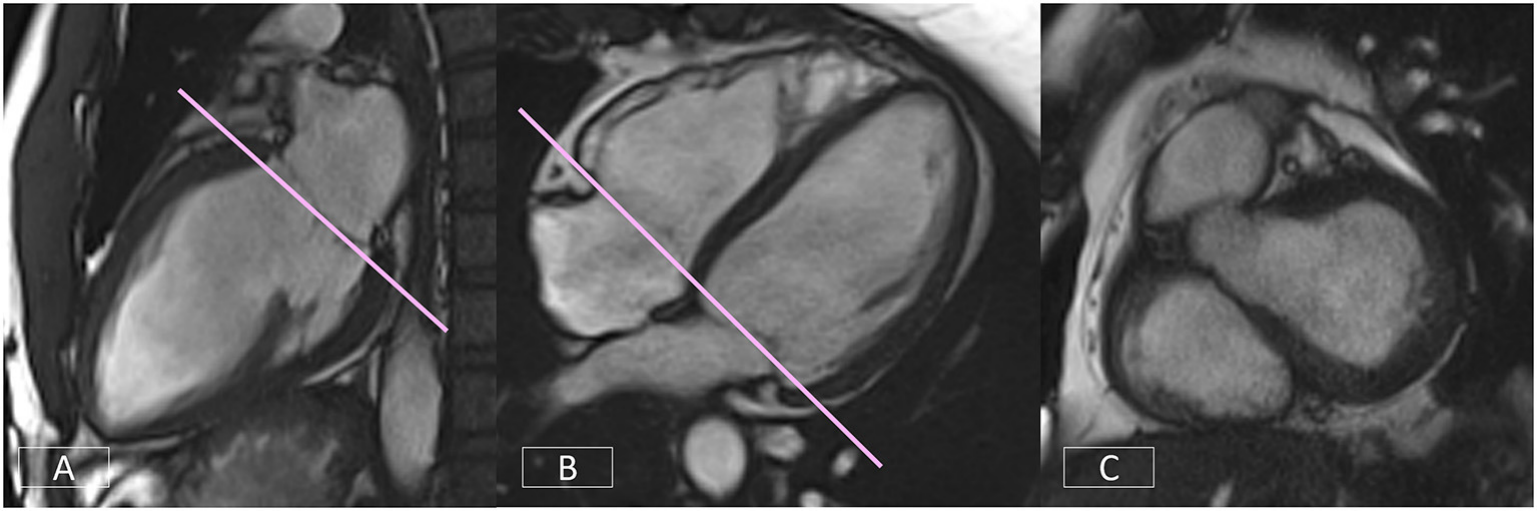
Accurate positioning of the basal slice of the LV SAX series requires the use of both the LV VLA **(A)** and the 4-chamber **(B)** views to ensure the basal diastolic phase slice is positioned parallel to the mitral valve annulus, avoiding atrium and with an even amount of myocardium around the blood pool **(C)**.

Correct positioning of the basal slice of the LV SAX stack can significantly improve the accuracy and reproducibility of volumetric analysis. A consistent and reproducible method of positioning this slice is critical. As outlined in Section Clinical Cardiovascular MR: What do we See and why do we Need it?, both the LV VLA (Figure 9A) and 4-chamber (Figure 9B) views must be used to ensure the basal diastolic phase slice is positioned parallel to the mitral valve annulus, avoiding atrium and with an even amount of myocardium around the blood pool (Figure 9C).

**Figure 10:**
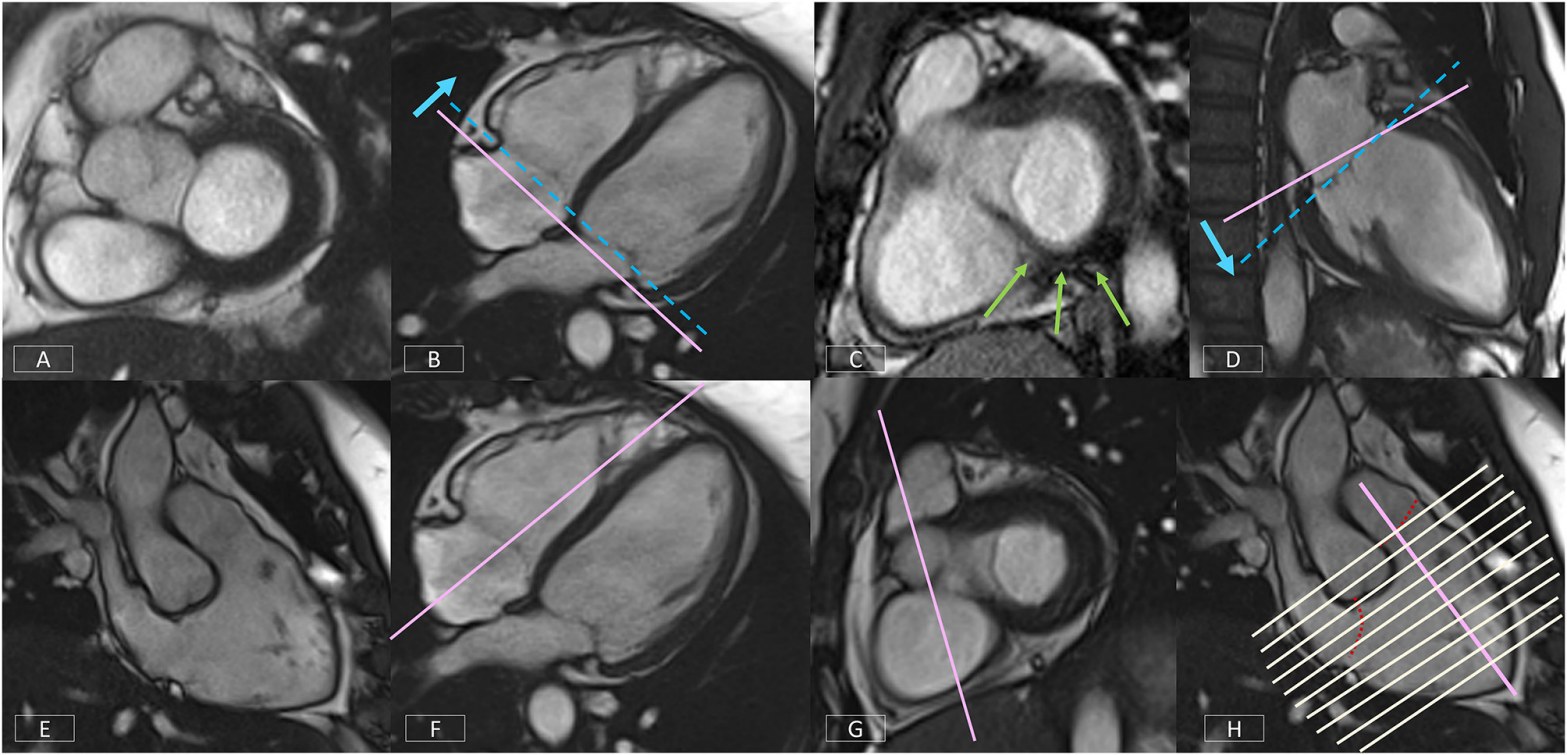
Top row: Positioning corrections for the LV SAX series include repositioning the slice more apically **(B)** if the basal diastolic phase slice includes atrium **(A)**. If there is an inconsistent amount of myocardium around the blood pool **(C)**, the slice angle is tilted on the LV VLA view **(D)**. Bottom row: A well-positioned RV VLA **(E)** is achieved by positioning the slice on the 4-chamber view **(F)** through the RV apex and avoiding the septum, then tilting the slice plane up to the RVOT and pulmonary valve on the basal LV SAX slice **(G)**. The RV SAX series can then be planned on this view to transect the tricuspid valve at an angle between 45° and 90° **(H)**.

If the image position is not correct, simple corrections are shown in Figure 10 (top row). If the basal diastolic phase slice includes atrium (Figure 10A), the slice must be repositioned toward the apex (Figure 10B). If there is an inconsistent amount of myocardium (Figure 10C), the slice angle is tilted on the LV VLA view (Figure 10D).

#### Right Ventricular Vertical Long Axis View

The non-geometric shape of the RV increases the complexity of positioning. A well-positioned RV VLA (Figure 10E) will enable visualization of the pulmonary and tricuspid valves, the RVOT and the RV apex in one plane. After positioning the slice on the 4-chamber view (Figure 10F) through the RV apex and avoiding the septum, the slice plane is tilted up to the RV OT and pulmonary valve using the basal LV SAX slice (Figure 10G).

#### Right Ventricular Short Axis

The modified RV short axis series (122) enables more accurate and reproducible planimetry of the ventricular borders making analysis less prone to operator error. A well-positioned RV VLA is key to ensuring correct positioning of the RV SAX.

Figure 10H shows the prescription of the RV SAX slices on the RV VLA. The slices should transect the tricuspid valve at an angle between 45° and 90° to ensure the slices are not prescribed too close to parallel to the valve.

### Building Blocks to a Comprehensive CMR Protocol

As mentioned earlier, the order of image acquisition is important for scanning efficiently. The operator should start by creating scan protocols of the building blocks outlined in Section The Building Blocks of a Successful CMR Exam. Using the template pulse sequence protocol created as per the facility requirements, each individual acquisition can then be built and named accordingly.

This foundation protocol then forms the basis of all the clinical protocols to be built on the scanner.

A general cardiomyopathy protocol can be used for the majority of clinical presentations. Options tailored to specific presentations, such as oedema-weighted imaging and T_2_-mapping for acute presentations, can be selected as required. Advanced imaging techniques, such as T_1_-mapping, should be added as appropriate (see Section Quantitative CMR Techniques). LGE imaging acquisitions should be built with plane specific labels, e.g., LV SAX LGE series, to assist in quickly identifying series when viewing images during reporting. Supplementary Table 1 is an example of a clinical protocol for the assessment of acute cardiomyopathic diseases such as acute myocarditis.

The next step is to build further indication-specific protocols matched to the facility clinical protocols, such as Hypertrophic Cardiomyopathy, Arrhythmogenic Cardiomyopathy or Tetralogy of Fallot where very specific clinical questions need to be addressed.

Using this method to build a comprehensive CMR protocol library will enhance efficiency, improve patient compliance, and ensure that all required imaging sequences are performed.

### Get Your Patient Ready

Performing an efficient CMR examination is highly dependent on patient cooperation. To optimize scanner time, the patient should be prepared outside the scan room. It is useful for patients to understand the important role they play in the quality of their examination, particularly the importance of consistent breath holding. Coaching breath hold procedures, checking breath hold capacity, and assessing likely compliance with instructions prior to commencing the examination will save valuable scanner time.

A good ECG trace is essential and is achieved by preparing the skin with abrasive gel, shaving if necessary and using low-impedance MRI safe electrodes. The use of an impedance meter to check electrode-to-skin contact and ensure strong lead voltage enables the operator to reposition using new electrodes prior to the patient entering the scan room. Once the patient is in the scanner and connected to the scanner gating system, the ECG trace should be assessed to ensure there is adequate voltage in each lead. If necessary, electrodes should be replaced and repositioned until a reliable ECG trace is obtained. If the poor ECG trace is due to the patient's irregular rhythm, acquisition strategies must be planned accordingly (see Sections Managing Challenging Patients and Handling Motion).

### Plan, Review, and Correct

Examination time can be reduced by attention to detail during scan preparation. With most CMR sequences requiring one breath hold per slice, it is important to limit the acquisition of any unnecessary slices. When acquiring a multi-slice series, each series should be prescribed carefully to cover only the anatomy needed. Images should be reviewed as they are acquired so that the series can be stopped if the anatomy is covered, rather than completing the full prescribed series.

Equally important is to observe the patient during each acquisition. Display the ECG and respiratory pulse on the console and be alert to patient movement; failure to hold breath for the full length of the scan; ectopic beats or irregular rhythms. It may be necessary to repeat slices with artifact, particularly if the images are part of a series used for quantitative analysis.

As a rule, the use of manual breath hold instructions improves patient compliance with breathing instructions and reduces the need to repeat slices due to breathing artifact.

### Managing Challenging Patients

#### Irregular Rhythms

Learning how to deal with irregular heart rhythms is one of the most important components of becoming a proficient CMR operator. Significant time can be lost if there is no management strategy in place. It is possible to achieve diagnostic images still within a reasonable timeframe by building protocols in advance with appropriate options for each pulse sequence type.

For each clinical protocol in the library, three acquisition strategies should be built: Sinus Rhythm, Mildly Irregular Rhythm, and Severely Irregular Rhythm (Supplementary Figure 4). Operators must learn when it is appropriate to change strategies and which strategy is required.

Retrospective gating should only be used for sinus rhythm or where there is a very occasional ectopic beat. The average heart rate range (RR interval) displayed on cine bSSFP images can be used to determine which strategy to use. Generally, if the variability is < ±40 ms, retrospective gating can be used (Sinus Rhythm strategy). Where the variability is greater, it will be necessary to change to prospective triggering for cine bSSFP and PC flow imaging (Mildly Irregular Rhythm strategy). Caution should be used when acquiring images used for quantitative analysis (such as parametric tissue mapping) to ensure the integrity and reliability of the data.

In the presence of a severely irregular rhythm, real-time and highly accelerated options will need to be employed (see Section Fast CMR: Speeding up Imaging by Acquiring Less Data).

#### Non-compliant Patients

For extremely unwell or claustrophobic patients, a plan for a short, high-value examination is required. Prior to commencing the examination, the clinical history and any previous imaging should be reviewed to determine the most critical clinical question. Generally, this will be information that no other diagnostic imaging test can provide such as tissue characterization. The imaging protocol is then planned to obtain this information as a priority in case it is necessary to terminate the examination prematurely. Protocols should be trimmed of any unnecessary sequences. For example, if the main question is the presence of myocardial fibrosis, an option would be to inject the contrast prior to moving the patient into the scanner, obtain the LGE first, then acquire any other imaging possible in the remaining time.

### Quantitative CMR: It Is Your Responsibility

CMR is highly operator and patient dependent and the quality of images obtained directly affects the accuracy and reliability of quantitative data. Surgical, therapeutic, or prognostic decisions are made on this data and attention to detail at every stage of the CMR examination is necessary to ensure the integrity of the results. It is incumbent upon the operator to recognize and report the limitations of the data if necessary.

## CMR Image Quality: No Free Lunch

**Figure 11:**
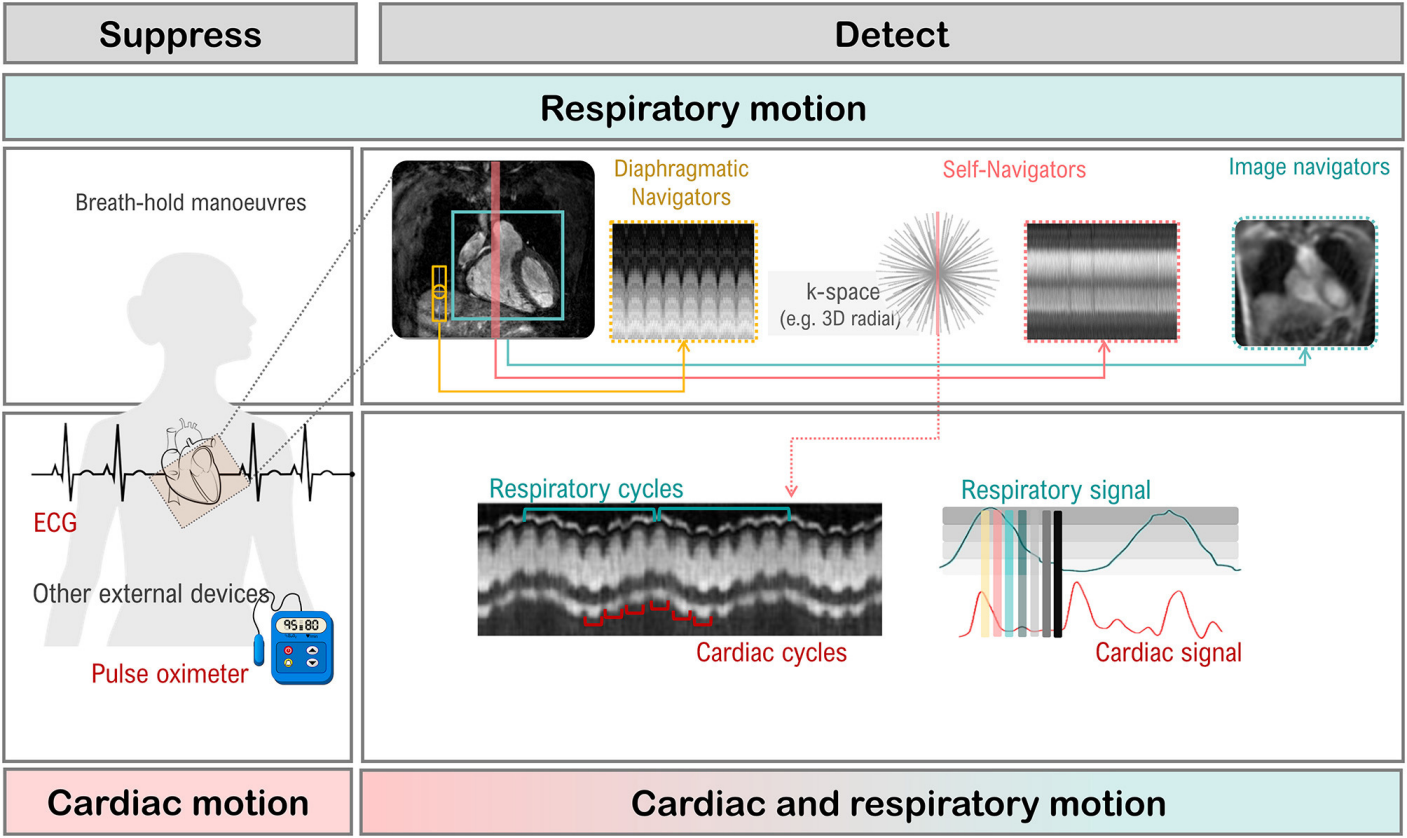
Cardiac and respiratory motion monitoring. Motion can either be suppressed (e.g., breath-holding) or monitored with MR navigators or external devices like electrocardiogram (ECG). From the monitored signal, one can extract the respiratory, and cardiac cycles which are needed for triggering (prospective) or gating (retrospective).

When setting up and optimizing a clinical CMR protocol to obtain the best images possible, the inherent trade-off between spatial and temporal resolution, scan time and signal-to-noise ratio (SNR) must be taken into consideration. For example, imaging at higher spatial resolution will result in lower SNR or longer scan times. Thus, a compromise in this triangle needs to be found in terms of image quality and acquisition time. Moreover, CMR can be impacted from image degradation due to cardiac and respiratory motion. Physiological motion induces aliasing along the phase-encoding direction and/or blurring of the image content (see Section Image Acquisition: What is the Position?), where the appearance depends on the imaging trajectory. Therefore, CMR acquisitions generally require synchronization or handling of the cardiac and respiratory cycles as depicted in Figure 11. In CMR, to avoid artifacts related to cardiac motion, it is usually desirable to freeze the heart motion, using gated/triggered acquisitions with <100 ms temporal resolution. Unfortunately, this comes at the expense of spatial resolution and/or coverage adding further constraints to CMR.

### Handling Motion

**Figure 12:**
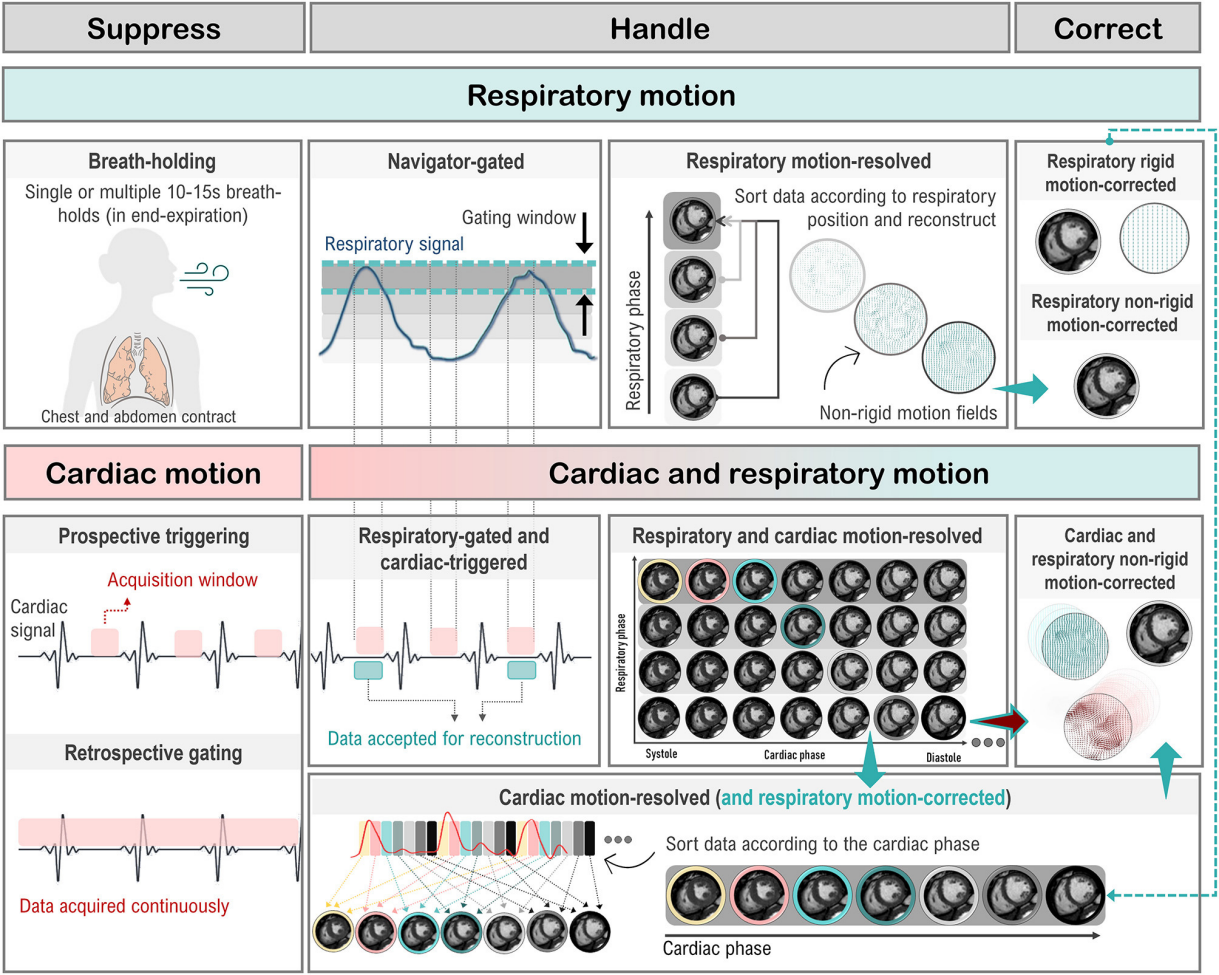
Cardiac and respiratory motion handling. Motion can either be suppressed (left column), handled prospectively or retrospectively (middle columns) or corrected/compensated (right column). Different strategies exist to deal with respiratory-only (top), cardiac-only (bottom left), and respiratory and cardiac (bottom right) motion. Prospective triggering: motion can be triggered to shorten the acquisition window to a specific motion state. Retrospective gating: motion is resolved by gating which can be performed exclusively on either respiratory/cardiac motion or on the joint respiratory and cardiac motion (central gating matrix) to yield respiratory/cardiac motion-resolved data. Data between individual gates/motion states can furthermore be compensated by registering them with a rigid or non-rigid motion field along the respiratory or cardiac motion direction.

Motion artifacts can be mitigated by (a) avoiding motion, i.e., training the patient to perform breath-holds or applying anesthesia and sedation to freeze respiratory motion; (b) reducing motion, i.e., signal averaging to smooth out motion, performing fast imaging to become less sensitive to motion (123–127) or suppressing motion outside the field of view using saturation bands; (c) triggering or gating motion, i.e., monitoring the motion cycle [using, for e.g., MR navigators (128–132), cameras (133), field probes (134), pilot tone (135), respiratory belts or electrocardiogram (136)] and either prospectively trigger on the respective motion (137, 138), meaning only acquiring within a small portion of the motion cycle, or retrospectively gate the motion (139–142), meaning sorting the data into distinct motion states for reconstruction. Motion avoidance [case (a)] requires, however, patient compliance and reduces patient comfort. For highly non-compliant patients (for e.g., pediatric patients), moderate sedation or general anesthesia can be given which does however require the use of a lung respirator, increasing scan time and costs, and could have potential side effects and complications. Motion reduction [case (b)] can require longer scan times, increases induced radio frequency energy on patient (i.e., tissue heating) and residual motion artifacts can remain in the image. Motion triggering and gating [case (c)] capture only a fraction of the entire dynamic respiratory and cardiac cycle or periodic assumptions of the dynamic cycle are made which may not hold in practice. Thus, a varied range of strategies has been proposed to avoid CMR image degradation due to cardiac and/or respiratory motion, some of which are summarized in Figure 12.

#### Respiratory Motion: You Can Breathe Normally

*Breath-holding* techniques are commonly used to reduce respiratory motion artifacts. If the patient complies with the breathing instructions this provides a 10–15 s window where artifact-free images can be obtained. A CMR examination requires multiple breath holds (143), which can lead to patient discomfort and fatigue, resulting in poor breath-holding and, consequently, motion artifacts that can impact the downstream analysis (144, 145). In addition, breath-holding can be challenging or impossible for pediatric, critically ill, or uncooperative patients (146). Moreover, some CMR protocols, such as CMRA and other 3D CMR applications, require acquisition times that are too long for a breath-hold. Free-breathing alternatives that use respiratory triggering or gating based on *diaphragmatic navigators* (that monitor the superior-inferior motion of the diaphragm) are available on most CMR scanners (147–149). Unfortunately, this approach has low scan efficiency, since only data within a small predefined respiratory gating window is used to generate an image, which leads to long and unpredictable scan times (due to irregular breathing patterns). External respiratory monitoring devices, such as bellows around the chest or abdomen, are also often used. More recently, novel tracking devices like pilot tone (135, 150) are being investigated for the usage of a sequence-independent motion monitoring solution.

Free-breathing CMR techniques based on *self-navigation* (151–155) or *image navigators* (130, 156–159) have been proposed to achieve 100% respiratory scan efficiency (no data rejection), by correcting all data for respiratory motion. Thus, allowing for shorter and more predictable scan times. Respiratory self-navigation techniques derive the respiratory-induced motion of the heart directly from the imaging data. Self-navigation is achieved by periodically imaging the central points in k-space and thus do not require any additional interleaving of navigators into the sequence. Typically, self-navigation approaches extract the respiratory signal from 1D projections of the field of view (in one or more directions). However, signal from static structures, such as chest wall, is also included in 1D self-navigators, which can lead to motion estimation and correction errors. Image-based navigators, which allow separation of static structures from the moving heart, have been proposed as an alternative to 1D self-navigation to reduce motion estimation errors. These methods use low spatial resolution images acquired with sequence interleaved imaging blocks at periodic intervals, prior to the CMR data acquisition, to estimate and correct for 2D or 3D respiratory motion. Free-breathing single shot CMR sequences often rely on retrospective motion correction using image registration methods to correct for respiratory motion between time frames.

Once the respiratory signal has been estimated, image degradation caused by respiratory motion can be reduced by: (a) correcting for translational motion (directly in k-space) (55, 130, 150, 158, 160–163), (b) separating (or binning) the data into multiple respiratory states to generate respiratory motion-resolved images (164–183), and (c) (using the latter for) correcting for more complex non-rigid motion (113, 157, 184–201).

#### Cardiac Motion: Stop Being Triggered

CMR acquisitions are usually synchronized with heart motion though an ECG (with electrodes attached to the chest) to minimize imaging artifacts caused by cardiac motion. Two approaches are typically used: prospective ECG triggering and retrospective ECG gating. *Prospective triggering* uses the R wave from the ECG signal to trigger the data acquisition (and “freeze” the heart) at a specific phase or certain number of phases of the cardiac cycle (149, 161, 202–206). In *retrospective gating*, data are acquired continuously throughout the cardiac cycle and the ECG signal is recorded simultaneously (140, 143, 144, 207–213). Subsequently, data are reordered and grouped into different cardiac phases according to the ECG signal. However, the ECG can be unreliable in CMR (as described in Sections Clinical Cardiovascular MR: What do we See and why do we Need it? and Clinical Cardiovascular MR: How Should we Perform the Examination), particularly in the case of arrhythmias and ectopic hearts. Finger pulse oximetry can be used as an alternative to ECG, but its signal is delayed relative to the ECG R wave. To overcome these challenges, cardiac *self-gating* approaches have been proposed to estimate an ECG-like signal directly from the acquired data (141, 142, 167, 171). The signal is then used for cardiac gating. More recently, contactless external sensors like pilot tone have also been used to track motion during CMR exams (214).

#### Cardiac and Respiratory Motion: No Stopping Now

Several solutions have been developed to eliminate the need for ECG synchronization and breath-holding altogether. This allows continuous acquisition of CMR data, known as *free-running CMR* (55, 167, 169, 170, 179, 197, 215). After acquisition, data is then sorted into multiple cardiac phases (with the desired temporal resolution) and multiple respiratory motion phases based on the cardiac (ECG, self-navigation, pilot tone, etc.) and respiratory (self-navigation, belt, etc.) motion signals to generate a multidimensional dataset for reconstruction. Moreover, the (self-navigation) respiratory signal or, for each cardiac phase, the bin-to-bin (affine) respiratory motion can be estimated and used to correct for respiratory motion directly in k-space (by applying the corresponding phase-shifts in k-space), before the image reconstruction, to generate respiratory motion-corrected cardiac phase-resolved CMR images (170, 182). In addition, these images can be used to generate cardiac motion-corrected images by selecting the cardiac phases with the smallest intra- and interphase motion and then correcting for non-rigid motion (200). The obtained respiratory and/or cardiac motion-gated k-spaces are usually sparsely sampled. During reconstruction, the spatio-temporal information can be exploited by either regularizing the motion dimensions (171, 179), correcting for the motion (216–218) or exploring the low-rankness (see Section CMR Reconstruction: From k-space to image space) of the dynamic processes (219, 220).

*Retrospective gating* assumes a periodicity of the temporal motion evolution which is however not a given for patients with arrhythmias or irregular breathing patterns (221, 222). In these cases, real-time CMR which is based on fast imaging sequences, like spGRE or bSSFP, can provide a viable solution (154, 157, 185, 222–228). Imaging with high (sub-second) temporal resolution makes acquisitions robust to motion, and thus, images can be obtained without gating or binning (220). In combination with efficient sampling trajectories and reconstruction techniques, 2D and 3D imaging with high spatio-temporal resolution can be performed.

### Fast CMR: Speeding up Imaging by Acquiring Less Data

Several approaches have been proposed to speed up CMR acquisitions by reducing the amount of data required for accurate reconstruction, including *parallel imaging* (125, 126), *k-t accelerated imaging* (173, 174, 229, 230), or pseudo-random sub-Nyquist sampling (123, 124, 231). Besides more efficient sampling trajectories, fast imaging sequences like fast low angle shot magnetic resonance imaging (FLASH) (232), bSSFP (24), fast spin-echo imaging (RARE) (233), echo planar imaging (EPI) (234) have enabled fast CMR imaging. Accelerated scans can be used to shorten the imaging time, to shorten breath-holds and improve patient comfort, but can also be used to collect more information (within the same imaging time), to increase temporal or spatial resolution and/or volumetric coverage.

*Parallel imaging* methods, such as (the image-based) SENSitivity Encoding (SENSE) (126) and (k-space-based) GeneRalized Autocalibrating Partial Parallel Acquisition (GRAPPA) (125), are used worldwide for CMR applications, but are limited by the number of receiver coils (see Section k-space) and in practice typically to 2- to 3-fold acceleration. *K-t accelerated imaging* (173, 174, 229, 230) extends these concepts along the dynamic temporal direction. It uses a regular undersampling pattern that is shifted over time. Images are reconstructed using a linear reconstruction approach, which relies on information extracted from low spatial resolution calibration data (with high temporal resolution) to minimize fold-over artifacts.

*Simultaneous multi-slice* (SMS) imaging (235–240) has the potential to acquire multiple slices, i.e., increasing cardiac coverage without sacrificing in-plane spatial resolution. However, pre-calibration scans are required to calibrate the unfolding during reconstruction which increase overall scan time.

On the other hand, reduced spatial coverage but increased dynamic resolution can be obtained with *real-time CMR* (154, 157, 185, 222–228). It relies on fast imaging sequences and trajectories to provide respiratory and cardiac motion-resolved images. Data acquisition is performed under free-breathing with sufficiently fast enough trajectories to capture whole field of view with minimal motion impact.

High acceleration factors can be achieved if the compressibility (or sparsity in a transform domain) of images is exploited as proposed in *Compressed Sensing* (CS) (124) or *Low-Rank* methods (178, 231). In these cases, we seek a (pseudo-) random sub-Nyquist sampling (i.e., undersampling) of the data. The applied sampling induces incoherent noise-like aliasing artifacts in the sparse domain. Thus, to satisfy the incoherence criterion (pseudo-) random Cartesian or non-Cartesian undersampling schemes are used to accelerate scans.

### CMR Trajectories: It Is That Sample

**Figure 13:**
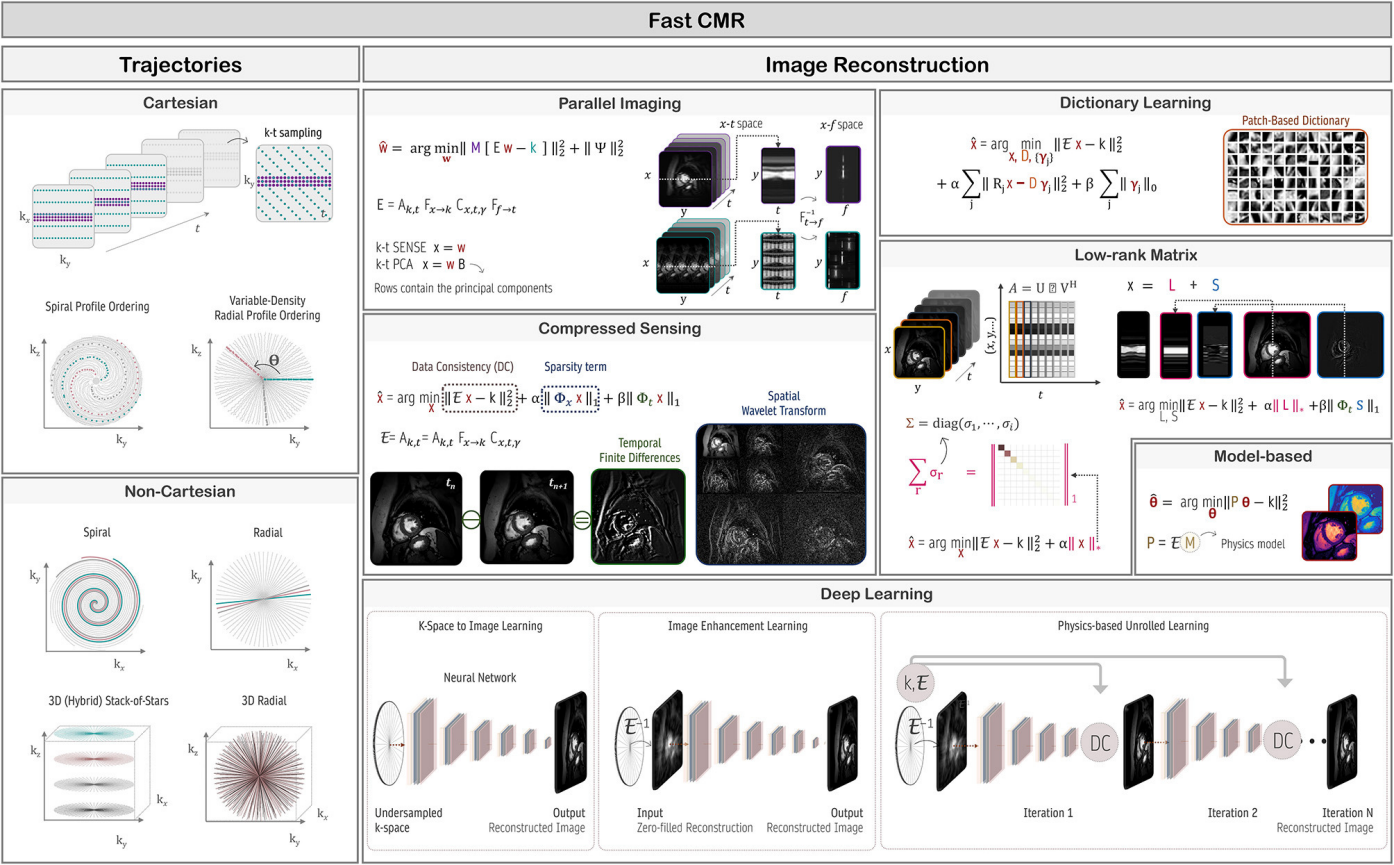
Fast cardiovascular MR techniques to enable high spatial and/or temporal resolved data acquisition. Cartesian or non-Cartesian undersampling trajectories (left column) can be used to accelerate acquisitions. Depending on the CMR application and acquired trajectory, various image reconstruction techniques (right column) like parallel imaging, compressed sensing, dictionary learning, low-rank, model-based, or more recently deep learning methods can be used. These reconstructions handle and exploit the spatial, temporal, and/or parametric dimensions. In CMR, the forward model, commonly given by k = Ex, maps the unknown (MR signal intensity) image series x to the k-space data k. The forward operator E contains the coil sensitivity maps C (enabling parallel imaging), Fourier operator F and sampling pattern A. If data are undersampled, dynamic images can be estimated using, for e.g., compressed sensing, by minimizing an objective function with a data consistency term (to enforce consistency between the measured data and model prediction) and a regularization term, with sparsifying transform Φ (e.g., spatial wavelet or total variation) and regularization parameter λ. Alternatively, a dictionary learning-based method can learn the sparsifying transform (dictionary, D), and reconstruct the image simultaneously from undersampled k-space data. The low-rank plus sparse (L + S) decomposition model enables the reconstruction of undersampled dynamic k-space data. In this case, the low-rank (L) component captures the temporally correlated background, and the sparse (S) component captures the dynamic information. Model-based reconstruction methods include the physics model in the forward model to directly estimate quantitative parameter maps from fully-sampled or undersampled k-space data.

The k-space undersampling patterns to accelerate CMR acquisitions in combination with the selected reconstruction method determine the obtainable image quality. A few exemplar trajectories are shown in Figure 13. In parallel imaging, the number of k-space lines is usually reduced using regular Cartesian undersampling (i.e., sampling below the Nyquist-Shannon sampling limit) (125, 126). In dynamic CMR, the Cartesian sampling patterns can be extended along the dynamic motion direction as used in k-t imaging (173, 174, 229, 230). A different k-space undersampling should be used for each time frame to introduce incoherence along the temporal dimension, and to thus enable exploitation of both spatial and temporal sparsity, as for example performed with a variable-density incoherent spatiotemporal acquisition (VISTA) sampling (241).

Non-Cartesian sampling schemes may be preferred because they are less sensitive to motion (123, 151, 163, 169, 171, 172, 181, 198, 202, 210, 224, 230, 237, 242–253), due to a densely sampled low-frequency range and the repeated sampling of the k-space center enables the extraction of motion signals (self-navigation). Unfortunately, non-Cartesian sampling requires resampling of the acquired data onto a Cartesian grid, which is computationally expensive.

Several Cartesian trajectories that acquire data using a radial or spiral-like pattern on a Cartesian grid have been proposed to overcome the computational complexity of non-Cartesian trajectories, such as Variable-Density sampling and Radial view ordering (VDRad) (254), CIRcular Cartesian UnderSampling (CIRCUS) (255), (Variable-Density) Cartesian acquisition with Spiral Profile ordering (VD-CASPR, CASPR) (201, 207, 256), GOlden-angle CArtesian Randomized Time-resolved (GOCART) (257), rotating Cartesian k-space (ROCK) (146), centric reordering (211) or Enhancing Sharpness by Partially Reduced Subsampling Set (ESPReSSo) (258, 259) sampling.

For 3D CMR imaging, non-Cartesian trajectories can be combined with Cartesian sampling, as in, for example, radial stack-of-stars (123, 175, 212, 260, 261) or stack-of-spiral (262) sampling schemes. Alternatively, 3D whole-heart CMR can be achieved using 3D Cartesian trajectories (167, 208, 254, 255), or 3D non-Cartesian sampling patterns, such as radial “koosh-ball” (169, 182, 209) or spiral phyllotaxis (170, 181). Moreover, acquisitions often use a golden-angle ordering scheme for which consecutive k-space spokes are incremented by the golden angle (θ ≈ 111.25°) (263, 264), to achieve nearly uniform k-space coverage (also optimal for retrospective binning) and incoherence along both spatial and temporal dimensions.

### CMR Reconstruction: From K-Space to Image Space

The undersampled data requires appropriate reconstruction techniques to recover an aliasing-free image, as illustrated in Figure 13. The raw data is linked with the image via the forward model as stated in Equation (11). CS relies on non-linear reconstruction algorithms to reconstruct images from randomly (or pseudo-randomly) undersampled data (124). In CS, the undersampling trajectory should lead to incoherent, noise-like aliasing artifacts which can be corrected for if images can be sparsely represented in a set transform domain (e.g., wavelets). In contrast to fixing the transform domain, *dictionary learning* techniques (265) seek to find the sparsest image representation by learning the sparsifying transform specific to each type of application. CS has the advantage that it does not require any training data and can achieve high accelerations. It can also be combined with parallel imaging methods (266, 267). However, it depends on application specific hyperparameter optimization, and the iterative algorithms result in long reconstruction times.

*Low-rank matrix completion* methods have extended the idea of CS to matrices (178, 231). These explore the global or local (patches) correlations within CMR images e.g., along the temporal or multi-contrast dimensions (113, 178, 179, 189, 207, 231, 268–278). For dynamic CMR, low-rank methods can act as an implicit motion compensation for any residual motion (after prior triggering/gating) (177, 207). Some methods simultaneously enforce low-rank and sparsity constraints to separate the temporally correlated background and dynamic information in various CMR applications, such as dynamic contrast-enhanced CMR (231, 274, 277). Moreover, *low-rank tensor imaging* has been proposed for multi-dimensional CMR imaging (169, 195, 197, 219, 220, 272, 279–281). These methods explore the spatio-temporal correlations in all dimensions (spatial, contrast, cardiac, and respiratory motion) to generate multi-parameteric and motion-resolved CMR images, e.g., cardiac- and respiratory-resolved T_1_ and T_2_ maps. In addition, motion can be handled implicitly in the low-rank decomposition instead of performing a prior motion gating.

*Model-based reconstruction* approaches have also been proposed to accelerate quantitative CMR imaging (248, 249, 282–285). These methods incorporate the physics of the MR signal into the image reconstruction problem allowing for the direct reconstruction of quantitative maps from the undersampled CMR data, bypassing the intermediate steps of image reconstruction and pixel-wise model fitting. Furthermore, in model-based reconstructions the underlying respiratory and cardiac motion model can be accounted for. Explicit motion compensation can be performed by mapping image data along the temporal direction with the underlying motion model extracted from image registration (187, 216, 217, 284).

Fast reconstruction is essential in a clinical setting. However, non-standard and iterative reconstruction methods often suffer from high computational demands, long computational times and require careful tuning of the algorithm (regularization) parameters. Recently, deep learning-based solutions have been proposed to address some of these shortcomings and which will be covered in more detail in Section Image Reconstruction.

## Artificial Intelligence for Cardiovascular MR

**Figure 14:**
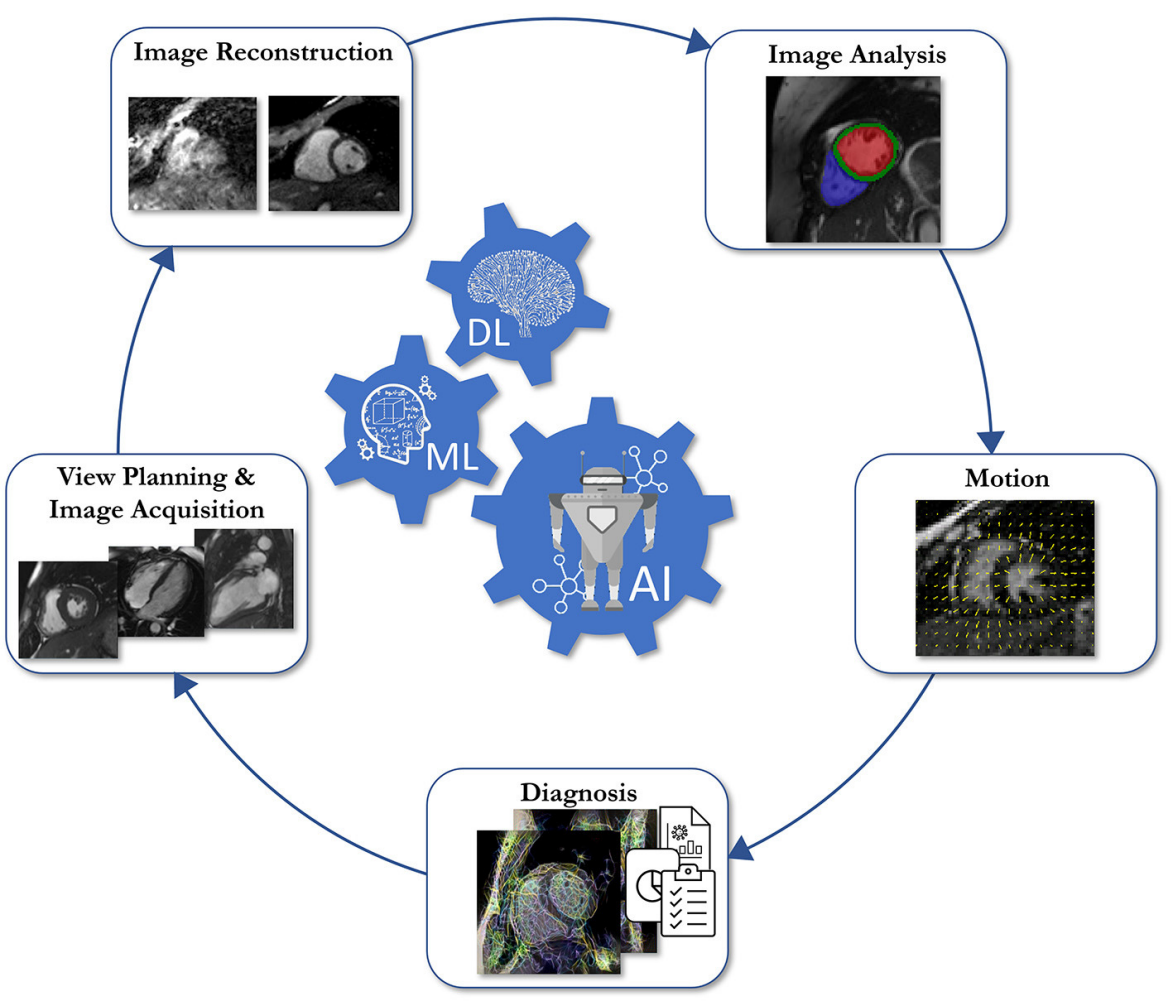
Schematic overview of the five areas in which Artificial Intelligence (AI)/Machine Learning (ML)/Deep Learning (DL) assisted operations can support the clinical workflow.

Artificial Intelligence (AI) and Machine Learning (ML), a sub-class of AI, have led to a break-through in the last years and have the potential to transform the clinical workflow substantially. CMR imaging leverages a high potential to enhance each individual step of the imaging pipeline (Figure 14), from complex CMR acquisition processes, the highly varying imaging protocols, to automated diagnosis. Despite the success of ML and AI, these new techniques should not replace clinicians, but aid clinicians in decision making, facilitate cardiac view-planning, or support in the tedious task of image segmentation to simplify and speed up quantification of functional cardiac parameters.

In this section, we first provide an overview of the common terminology and building blocks in ML, without the usage of complex mathematical notations. In the second part, we provide an overview on how ML can be used at each individual stage of the imaging pipeline, i.e., cardiac view-planning, image acquisition, image reconstruction, shape analysis, image segmentation, and quantification of biomarkers. Finally, we provide insights into potential pitfalls in using ML in CMR, and an outlook into the future of ML for CMR.

### Breaking Down the Terminology

The terms AI, ML and more recently deep learning (DL) are often used interchangeably. However, there is a huge difference between these terms. AI leverages the potential of machines to mimic the human mind's ability to solve problems or make decisions. As a sub-branch of AI, ML uses algorithms to learn patterns from data and make predication about a certain task. Pre-defined features are extracted from the input data and are then fed into the (statistical) model. The parameters of this model are then trained using data to make correct predictions for a specific task without human assistance. After model training, predictions from new unseen data can be made using the trained model parameters. While ML in its classic formulation depends on defining hand-crafted discriminative features which are tedious and time-consuming to extract, a further sub-branch of ML called DL directly learns feature representations from data using neural networks (NNs). Although the concept of NNs was established in the late 1980's, DL has flourished since 2015. The break-through of DL came with the availability of Graphics Processing Units (GPUs), large datasets, and advanced architectures.

ML has the ability to support in various challenging tasks. In image *classification*, the model takes the input image or already extracted features as input, and outputs a classification label, to predict, e.g., a certain heart disease. The task of assigning an individual label to each input pixel is called *segmentation*. Typical application of image segmentation in the field of cardiac imaging is the segmentation of the heart into four chambers and myocardium. Image-to-image or sensor-to-image translation describe regression tasks that form the third group of important ML tasks. *Regression* tasks can be found in MR image reconstruction from undersampled k-space data, super-resolution, or image enhancement.

### Types of AI: Does It Need Supervision?

Machine Learning can be categorized in three major types: supervised learning, self-supervised learning, and un-supervised learning. Supervised learning methods require a training database with a set of input data and annotated output labels for training. The model tries to make predictions, which are then compared to the correct output labels using a cost function. The error in the cost function then gives an idea how the models' parameters have to be updated in the training loop. In contrast, neither labeled data nor any other prior knowledge on the data is available in unsupervised learning. Hence, the model learns itself how to identify patterns in the data, as in clustering (variational) autoencoders, or Generative Adversarial Networks. Self-supervised learning is a form of unsupervised learning, where the data provides the supervision.

### Training, Validation, and Testing: Getting It to Work

To update the parameters of the model, the network needs to be *trained* with respective training data. A training database consists of a number of training samples. For each update of the model parameters, a *batch* is drawn from the training samples and passed through the network. This is repeated until all training samples have been processed, defining one *epoch* of training. The network itself is trained for several epochs until convergence. The number of training epochs depends on the selected dataset, the number of training samples, and the selected task. A separate *validation* data set is used to monitor the training process and to tune hyper-parameters (learning rate, architecture parameters etc.). This allows for, e.g., identification of model overfitting. However, the validation data set is not used to update the model parameters of the network. In the *testing* stage, the model is tested on further unseen data and used for the final model evaluation. It is important to note that training data, validation data, and test data are mutually exclusive.

However, in medical imaging, and especially in the context of CMR, only small databases are often available. This requires a thorough study of the robustness of the model to avoid a bias toward selected validation samples. Cross validation provides a way to study the robustness of models if small datasets are available. For k-fold *cross validation*, the database is split into a number of k subsets and the networks are trained for training data in k-1 folds, and the data in the remaining fold is used for validation/test. This is repeated such that k networks are trained, with every fold being used for validation. Deviations in evaluation metrics indicate reduced robustness of the models.

In medical imaging, we often observe another danger when creating our own training databases. In CMR, we often acquire several slices from a single subject. Hence, in case training samples are drawn from multiple subjects with several slices each, we need to make sure that data from the same patient does not appear in training and validation simultaneously to avoid any bias of the models toward specific anatomies or pathologies.

### Database: Does Size Really Matter?

The availability of large training databases is one of the most challenging aspects in ML for CMR. In the context of image reconstruction, publicly available datasets are very rare. In CMR, for example, we found that some data for radial image reconstruction of dynamic cardiac MRI are available (286). For other ML tasks like image classification and image segmentation, tens of thousands of annotated CMR datasets are available in, e.g., UK Biobank (287), M&M (288), or ACDC (289). However, the availability of both raw k-space data for image reconstruction and annotations of the same data for image segmentation or cardiac disease classifications are still limited. Hence, in the context of multi-task models, special focus must be given on the datasets, as k-space data for image reconstruction tasks are often simulated or only retrospectively undersampled, limiting the application of proposed approaches to clinical workflow.

### Neural Networks and Their Building Blocks: How to Build Your CMR Network From Scratch

The recent success of neural networks not only depends on the availability of training data, but also on the availability of expressive network architectures. The idea of neural networks goes back to the 1957, where an artificial neuron was modeled similar to the neurons in the brain (290). An input signal arrives at the neuron (layer) and is processed by the layer weights. An activation function decides if the neuron should be fired or not. A typical deep neural network consists of several layers, which can be related to modeling the complex wired structures in the brain.

*Convolutional layers* are powerful local feature extractors. The spatially-dependent features are generated by convolving the underlying image with a set of trainable filter kernels, optimized during model training. To extract global features, *fully connected layers* are used, connecting each input pixel with each output pixel. Global features are necessary in, e.g., image classification and segmentation. To emphasize the extracted features, non-linear *activation functions* are applied. Common activation functions for image regression are ReLU and its various variants, e.g., LeakyReLU, PreLU, while for image segmentation and classification tasks bounded activation functions such as tanh, sigmoid, or softmax are used. *Pooling layers* or *strided convolution layers* are used to downsample the spatial features, to increase the receptive field. To increase the resolution, *strided transposed convolution* layers or *upsampling layers* that perform interpolation are common choices. In-between convolution and activation layers, often *normalization layers* are used that are reported to stabilize training and improve training convergence. These are the basic elements to build a deep neural network, however, more detailed building blocks are out of scope of this review paper.

It should be noted that the nature of MR data is complex-valued, but in the core literature only real-valued building blocks are reported. Hence, the real and imaginary plane are handled as a real-valued image with two feature channels. Recent works also focused on the implementation and correct utilization of complex-valued versions of the aforementioned building blocks, and correct network training following Wirtinger calculus (291).

### CMR Applications

**Figure 15:**
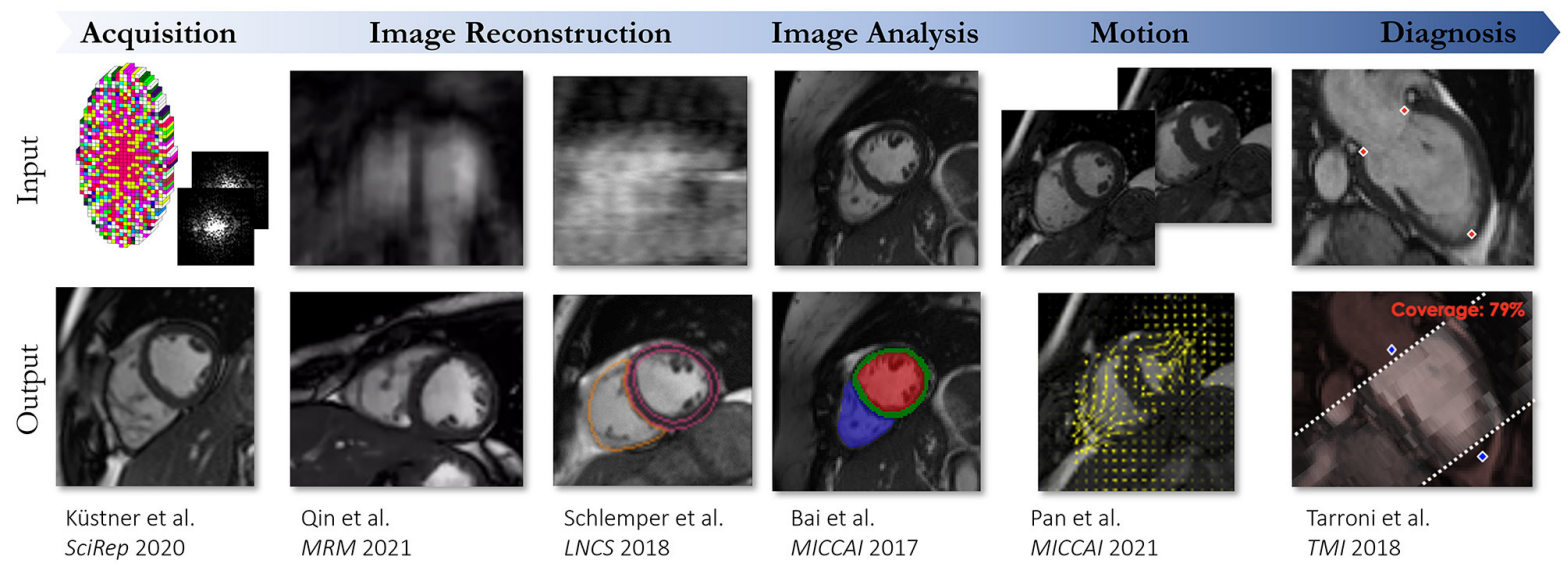
Exemplary AI-assisted applications performed on cardiac CINE MRI ranging from acquisition over image reconstruction, analysis, motion to diagnosis. The respective inputs and output data is illustrated.

The presented layers and building blocks can be used to form a full network. For CMR, convolutional neural networks are most used, however, no unique network definition exists, and numerous variants have been proposed for various tasks. The targeted application, dimensionality and data availability mainly determine the task definitions and subsequently architectural choices. An exemplary scenario for AI applications for cardiac cine MRI over the various CMR processing steps (Figure 14) is depicted in Figure 15.

### View Planning and Image Acquisition

A comprehensive CMR exam requires complex and time-consuming scan planning and optimization of acquisition protocols. Thus, the effectiveness and image quality of a CMR scan highly depends on the experience and ability of the operator to adequately prepare patients, tune acquisition parameters, plan cardiac views and shimming, all in a timely fashion (see Section Clinical Cardiovascular MR: How Should we Perform the Examination). Currently, highly trained operators manually plan and conduct clinical CMR exams. Recently, ML methods have been proposed to automate or shorten the scanning process, standardize image acquisition and quality across patients (292–296). Major CMR vendors are now introducing ML solutions to help or automatically optimize and plan exam protocols. Other ML methods have been proposed for assessing image quality, replacing the usually subjective visual inspection, which can detect artifacts, correct acquisition parameters, and trigger a rescan if deemed necessary (297–299). Moreover, ML methods can also be used to learn the optimal sampling pattern for reducing the acquisition time while maximizing image quality (300). Thus, ML-assisted CMR examinations can help operators solve complex decision-making tasks under time pressure.

### Image Reconstruction

In MR image reconstruction, we aim at recovering an image from (undersampled) k-space which is corrupted by measurement noise. The acquisition process is thereby approximated and formalized in a linear forward operator, see Equation (11). Depending on the imaging application and signal modeling, the operator involves Fourier transforms, sampling trajectories, and coil sensitivity maps. Field inhomogeneities, relaxation effects, motion, and diffusion can also be considered.

In ML frameworks, the objective is to learn the sensor-to-image mapping function having learnable parameters. The mapping function can be stated as NNs and can be used in different ways to reconstruct an image from the measured k-space data. All tasks have an image and/or k-space as input but differ in how data is processed and how further MR specific information (meta parameters or other tensors like trajectories and coil sensitivity maps) are handled for the targeted application output. These reconstruction tasks are further described hereafter and depicted in Figure 13.

#### Image Enhancement Learning

Certain types of undersampling introduce incoherent noise-like aliasing in the zero-filled reconstructed images. Thus, an image enhancement task can be used to reduce the noise-like aliasing in the images. The network performs an image-to-image regression by predicting the output value based on the corrupted input image. The input to the denoising task can be the zero-filled (and noise-affected) MR images or reconstructed MR images that present remaining aliasing or noise amplification for high undersamplings (e.g., images reconstructed with parallel imaging). Instead of learning the denoised image, some approaches learn the residual noise to be removed from the noisy input (301–303). The mapping only acts on the image and does not consider any further information from the acquired k-space. Hence, data consistency to the measured k-space signal cannot be guaranteed. Approaches exist that add additional k-space consistency to the cost function (304) or enforce k-space consistency after image denoising (305, 306).

#### K-Space to Image Learning

A different DL-based approach is to reconstruct the MR image directly from the acquired k-space data. With the so-called direct k-space to image mapping, the k-space data are directly used as the input. Consequently, the network approximates the forward model (see Section CMR Reconstruction: From K-space to Image Space). Learning a direct mapping is especially useful if the forward model or parts of the forward model are not exactly known. In the case of fully sampled MRI under ideal conditions, the learned mapping approximates the Fourier transform (307). However, this becomes computationally very demanding due to fully connected layers which are involved here. Furthermore, consistency with the acquired k-space data cannot be guaranteed.

#### Physics-Based Unrolled Learning

Another family of DL-based MR reconstruction methods is referred to as physics-based reconstruction. These approaches integrate the traditional physics-based modeling of the MR encoding (see Section K-space) with DL, ensuring consistency with the acquired data. We can distinguish two classes of problems: (a) learning in k-space domain and (b) iterative optimization in image domain with interleaved data consistency steps. The first approaches are referred as k-space learning whereas the latter one is known as unrolled optimization. These two approaches can be combined to hybrid approaches that learn both a neural network in k-space and image domain.

##### K-Space Learning

A prominent approach for physics-based learning in k-space domain (308) can be viewed as extension of the linear kernel estimation in GRAPPA. A non-linear kernel modeled by the network is learned from the ACS. The missing k-space lines can then be filled using this estimated, non-linear kernel and the data is then transformed to the image space using an Inverse Fourier transform. The final image is obtained by root-sum-of-squares reconstruction of the individual coil images.

##### Hybrid Learning

Hybrid approaches (309–311) combine the advantages of learning in k-space and image domain. These networks are applied in an alternating manner to obtain the final reconstruction. When designing hybrid approaches, it is important to keep the basic theorems of the Fourier transform in mind: local changes in image domain result in global changes in k-space domain and vice versa, to avoid unexpected behavior.

##### Plug-and-Play Priors

Trained image denoisers can be also combined with physics-based learning or conventional iterative reconstructions and thus serve as an advanced regularization for a traditional optimization problem. Iterative, image-wise, or patch-wise denoising is performed followed by a subsequent data consistency step, as involved in plug-and-play priors (302, 312–314), regularization by denoising (315) or image restoration (316).

##### Unrolled Optimization

Physics-based learning, which is modeled as iterative optimization, can be viewed as generalization of iterative SENSE (126, 317) with a learned regularization in image domain. It contains a data-consistency term and a regularization term which imposes prior knowledge on the reconstruction. A gradient descent (318), proximal gradient (319), variable splitting (320), or primal-dual optimization (321) can be used for algorithm unrolling. The iterative optimization scheme is unrolled for a fixed number of iterations. Neural networks replace the gradient of the hand-crafted regularizer by a learned data-driven mapping. Various regularization networks can be used*, e.g.*, variational networks (322) or cascade of convolutional networks (319). In CMR, the dynamic or quantitative dimensions can be incorporated into network architecture design [e.g., recurrent networks (311, 323)], building blocks [e.g., 2D+t (324), 3D+t convolutions (325)], data priors (326), or loss modeling (327). Training several iterations with alternating mapping functions and intermittent data consistencies reflect thus unrolled optimizations (328).

#### Super Resolution

An alternative approach for accelerating the image acquisition while simultaneously increasing spatial resolution is the usage of DL-based super resolution (SR). Images are acquired at a low-resolution (with or without undersampling) and retrospectively reconstructed to the high-resolution target. This has been studied for cardiac cine (329, 330) and whole-heart CMR (331–336).

### Image Analysis

CMR image segmentation and quantitative evaluation can be a challenging, time-consuming and operator intensive task. However, quantitative analysis of myocardial function, perfusion, pathological tissues, provides important diagnostic and prognostic information (66). In recent years, a large number of ML-based methods have been proposed to automatically perform CMR image segmentation and analysis, thereby significantly reducing the time required for CMR image assessment (337). Considerable efforts have been directed toward cine imaging, as it is considered the gold standard for the assessment of cardiac chamber volumes and function (338–341). In this case, DL-based methods automatically segment the myocardium and cardiac chambers from MRI images, replacing manual approaches that are time-consuming and prone to observer variability, to enable the extraction of quantitative indices, such as LV and RV volumes, mass, and EF. Some frameworks additionally provide myocardial strain measures (299, 342). Automated segmentation methods have also been proposed to quantitatively derive other important markers of cardiovascular disease such as volume of pericardial adipose tissue (343), and scarred tissue areas (from LGE images) (344–348). Moreover, few DL-based methods have proposed to automatically quantify myocardial tissue from native T_1_ mapping (349, 350) and myocardial blood flow from contrast-enhanced perfusion CMR (351, 352).

### Motion Correction

Motion artifacts due to physiological motion or caused by mis-triggering in the ECG or movements during the examination are a potential source of image degradation. Several approaches using DL exist to correct for motion artifacts in the area of CMR.

Adversarial training strategies as proposed in Zhang et al. (353) aim to correct for the motion in the image domain. A database of motion-corrected and motion-degraded images serve as training database. A generator network predicts motion-corrected images. The discriminator network tries to distinguish if the generated motion-corrected image is from the manifold of real motion-corrected or generated motion-corrected images. The goal of the generator network is to fool the discriminator network to generate images that look like real motion-corrected images. Another method of retrospective motion correction in CMR with adversarial training is proposed in Ghodrati et al. (354). Here, a Variational Autoencoders is trained on healthy subjects and patients with suspected cardiovascular disease to remove respiratory motion.

Instead of addressing MR motion correction in the image domain, Oksuz et al. (355), apply motion correction directly in k-space. Their method uses a generator network that is motivated by Automap (307) to transform the k-space directly to a reconstructed image. Pairs of synthetically motion-corrupted k-space data and artifact-free reconstructed CMR images serve as training database for the proposed adversarial training strategy. Beyond image reconstruction, Oksuz et al. (356) introduced a joint framework for motion artifact detection and correction in k-space and image segmentation. The motion artifact network detects motion-affected lines in k-space, influencing the data consistency term. The motion-corrected image is obtained from a subsequent bi-directional recurrent CNN. The segmentation network is based on a standard UNet architecture (357). Their work showed that end-to-end training outperforms sequential training substantially when trained on UK Biobank data (287), with synthetical motion corruption and synthetically added phase information.

Retrospective motion-correction of reconstructed CMR images is proposed in Huang et al. (358). First, CMR reconstruction is performed with a Convolutional Gated Recurrent Units and a subsequent data consistency layer. Motion fields are then estimated from the reconstructed images using a FlowNet architecture. The estimated motion fields are then used in a post-processing motion-correction step to improve the final reconstruction.

Large non-rigid motion across multiple temporal frames can occur and in the case of 2D imaging, the existence of through-plane motion complicates the motion estimation process. A fast and reliable motion estimation is therefore required that correlates these short- and long-term correspondences, as proposed by Pan et al. (359) and Küstner et al. (360), and either operates on the image domain (359) or on the accelerated raw data (360).

Joint motion estimation and reconstruction is proposed by Seegolam et al. (361). Here, a motion-estimation UNet is embedded directly in the data consistency term of a dynamic reconstruction network. This approach allows for exploiting the whole temporal information in each cardiac phase, resulting in high-quality reconstruction of extremely undersampled CMR data.

### Multi-Task Networks

Sun et al. (362) proposed a unified deep network architecture for joint image reconstruction and segmentation. Image reconstruction is formulated as a cascaded encoder-decoder network with intermittent data consistency layers to facilitate learning and making use of the acquired k-space data. The reconstruction and segmentation networks share the same encoder, acting as a regularizer for the two tasks, while the decoder is different, and hence, task specific. Their results suggest that training a joint network is beneficial for high-quality segmentation of undersampled k-space data, however, the evaluation was performed on simulated k-space data of the MRBrainS segmentation challenge dataset (363). Similar observations were made in Huang et al. (364) where FR-Net for image reconstruction (inspired by the fast iterative shrinkage-thresholding algorithm), was combined with a UNet for myocardial segmentation. However, this approach was evaluated only on simulated k-space data. While these multi-task networks aim for a reconstructed intermediate image, Schlemper et al. (365) bypassed this step and directly predicted segmentation maps from highly undersampled dynamic CMR images of the UK Biobank data (287). Their results indicate that clinical parameters can be computed within an error of 10% if at least 10 lines are acquired for each cardiac phase, using Cartesian sampling.

Joint learning of motion estimation and segmentation from fully-sampled data was proposed by Qin et al. (366). An extension to undersampled data has been proposed in Qin et al. (367), where the network training is guided by fully-sampled data. The results suggested that an efficient motion estimation network can bypass the need for high-quality reconstructions in order to achieve accurate image segmentation.

The surveyed approaches achieve promising results for end-to-end training. However, to date, none of these approaches have been tested on real k-space data and evaluated for clinical applicability. Furthermore, evaluation of these k-space data also requires the availability of proper training databases with both real k-space data and manual segmentations.

## Challenges and Conclusion

The plethora of CMR sequences available and information offered makes the technique attractive, but also very challenging, particularly for a beginner. This review has provided an overview of the main CMR concepts and techniques, including recent technical advances, which should be useful for anyone wanting to improve, update, or maintain their knowledge and understanding of CMR. Ultimately, the dialogue between the scientific and clinical communities should improve if all users understand CMR terms and use a common language. This review has described the key physical principles underlying the most commonly used (quantitative) CMR sequences and preparation pulses and causes of common image artifacts. This review has explained how and why CMR can (and should) be used for diagnosis and guiding clinical decision making in a range of cardiovascular disease scenarios, such as ischemic heart disease, myocarditis, atrial fibrillation, valvular heart disease, vascular disease, congenital heart disease, and cardiac tumors. This review has also outlined the building blocks of a CMR examination, explained how to perform a comprehensive patient-tailored examination based on these building blocks in a clinically acceptable timeframe and avoid common scanning mistakes. The challenges of CMR associated with acquisition time, SNR, spatial and temporal resolution, cardiac and respiratory motion have been discussed. In addition, popular and recently developed methods of suppressing and handling motion have been described. This review has explained how to speed up CMR scans by acquiring less data (than needed by conventional methods) using (pseudo-)random sampling trajectories and non-linear reconstruction algorithms, such as compressed sensing and low-rank completion, model-based or DL reconstruction approaches. Finally, this review has discussed how DL approaches can potentially help overcome challenges such as time-consuming reconstructions and quantitative analysis.

## Author Contributions

TI, WS, SW, CC, MB-I, KH, TC, and TK wrote sections of the manuscript. TC and TK edited the sections and manuscript. All authors contributed to manuscript revision, read, and approved the submitted version.

## Funding

This work was supported by the Deutsche Forschungsgemeinschaft (DFG, German Research Foundation) under Germany's Excellence Strategy—EXC 2180 #390900677 and EXC 2064/1 #390727645. This work was part of a project that has received funding from the European Union's Horizon 2020 research and innovation programme under grant agreement No. 867450.

## Conflict of Interest

The authors declare that the research was conducted in the absence of any commercial or financial relationships that could be construed as a potential conflict of interest.

## Publisher's Note

All claims expressed in this article are solely those of the authors and do not necessarily represent those of their affiliated organizations, or those of the publisher, the editors and the reviewers. Any product that may be evaluated in this article, or claim that may be made by its manufacturer, is not guaranteed or endorsed by the publisher.

## Supplementary Material

Supplementary Figure 1 Still from a balanced steady-state free precession 4-chamber cine. This sequence has high intrinsic T_1_ and T_2_ contrast enabling cardiac chambers and anatomy to be visualized with exquisite clarity. The blood pool appears bright. Fat also appears high signal. Areas where fat and water protons interface appear black in outline due to chemical shift artifact. This allows fibrofatty change (arrows) to be readily visualized in the septum (arrows) and epicardial lateral wall, enabling a diagnosis of arrhythmogenic cardiomyopathy to be made without the need for contrast.

Supplementary Figure 2 Balanced steady-state free precession cine demonstrating a jet of aortic regurgitation (arrow). This cannot be reliably quantified by visual assessment of the jet and once detected should be further evaluated using phase-contrast velocity mapping.

Supplementary Figure 3 Quantification of aortic regurgitation in the patient depicted in Figure 9 using phase-contrast velocity mapping. The magnitude image on the top right is used to contour a region of interest in the aorta. The phase image on the top left is used to determine flow and plot this against time (bottom). The flow drops below the baseline for the whole of systole. The regurgitant volume is ~67 ml which amounts to a regurgitant fraction of 60%, denoting severe regurgitation.

Supplementary Figure 4 An example of a clinical imaging protocol library built with three acquisition strategies for managing irregular rhythms: Sinus Rhythm; Mildly Irregular Rhythm; Severely Irregular Rhythm.

Supplementary Table 1 A general cardiomyopathy protocol can be modified to assess acute presentations by the addition of imaging sequences sensitive to oedema (highlighted).
