# Supplementary figures and images for: Cardiac MR: From Theory to Practice
Source: Front Cardiovasc Med. 2022 Mar 3;9:826283. doi: 10.3389/fcvm.2022.826283 (PMC8927633; doi:10.3389/fcvm.2022.826283)

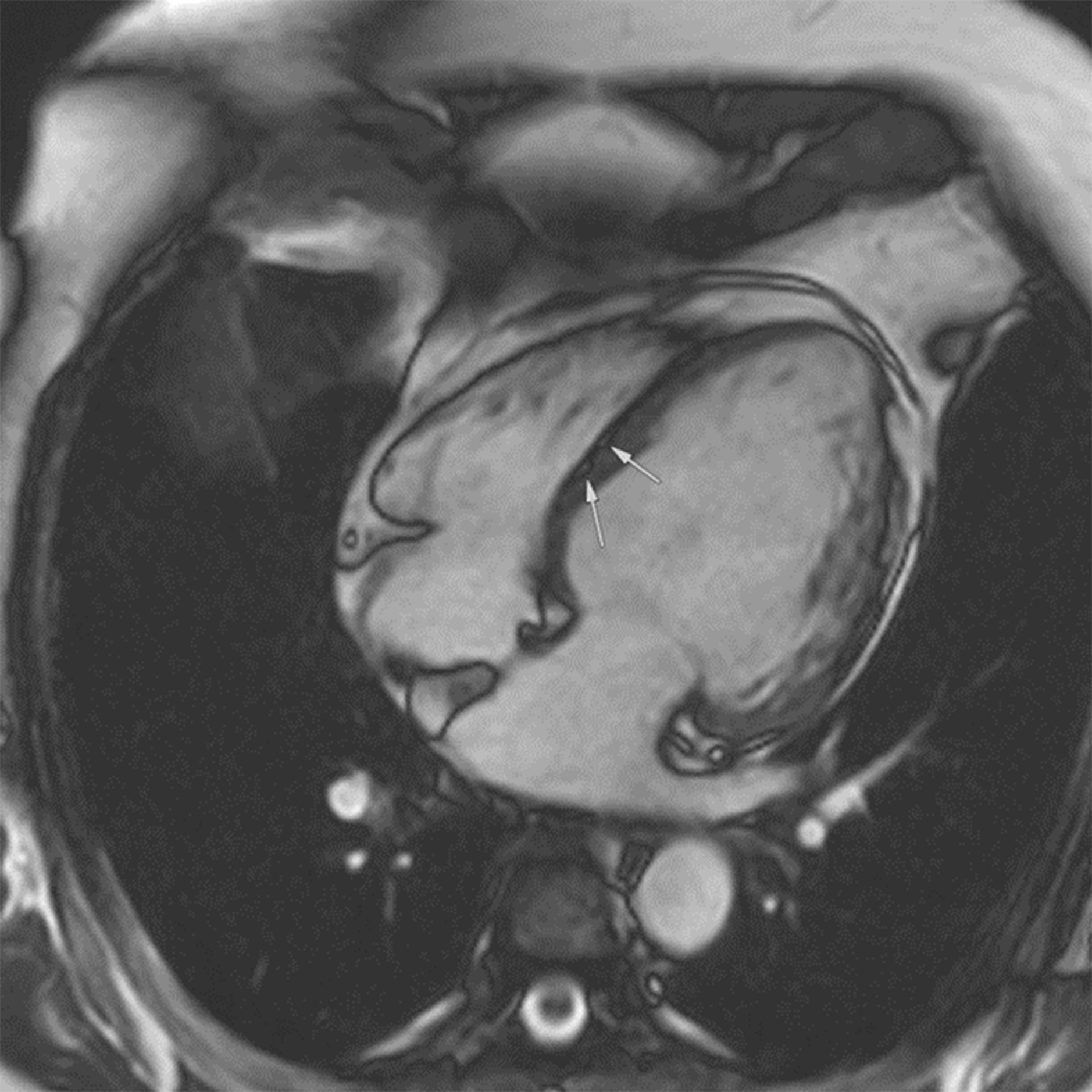

Supplement: Supplementary Figure 1 — Still from a balanced steady-state free precession 4-chamber cine. This sequence has high intrinsic T1 and T2 contrast enabling cardiac chambers and anatomy to be visualized with exquisite clarity. The blood pool appears bright. Fat also appears high signal. Areas where fat and water protons interface appear black in outline due to chemical shift artifact. This allows fibrofatty change (arrows) to be readily visualized in the septum (arrows) and epicardial lateral wall, enabling a diagnosis of arrhythmogenic cardiomyopathy to be made without the need for contrast. [file Image_1.PNG]

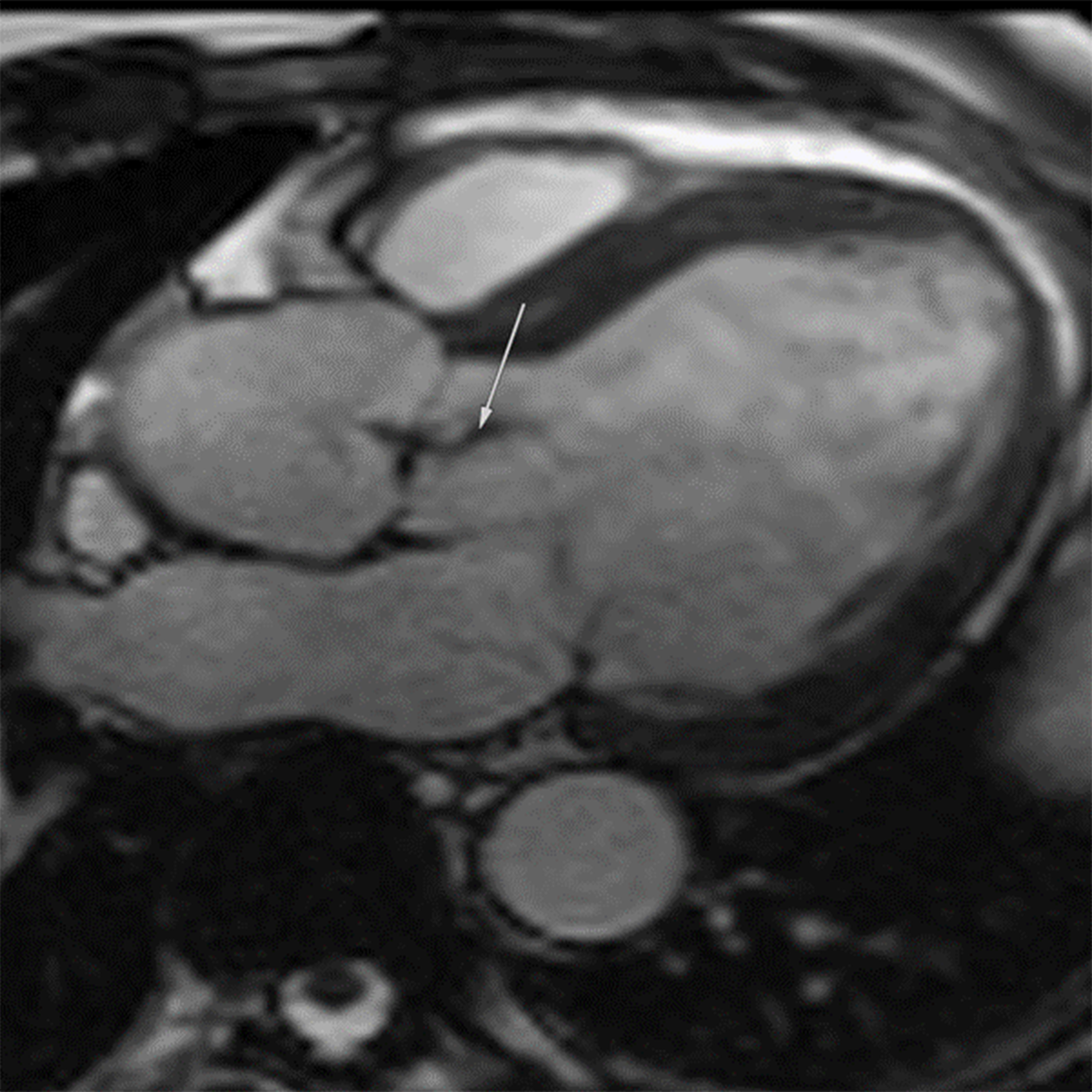

Supplement: Supplementary Figure 2 — Balanced steady-state free precession cine demonstrating a jet of aortic regurgitation (arrow). This cannot be reliably quantified by visual assessment of the jet and once detected should be further evaluated using phase-contrast velocity mapping. [file Image_2.PNG]

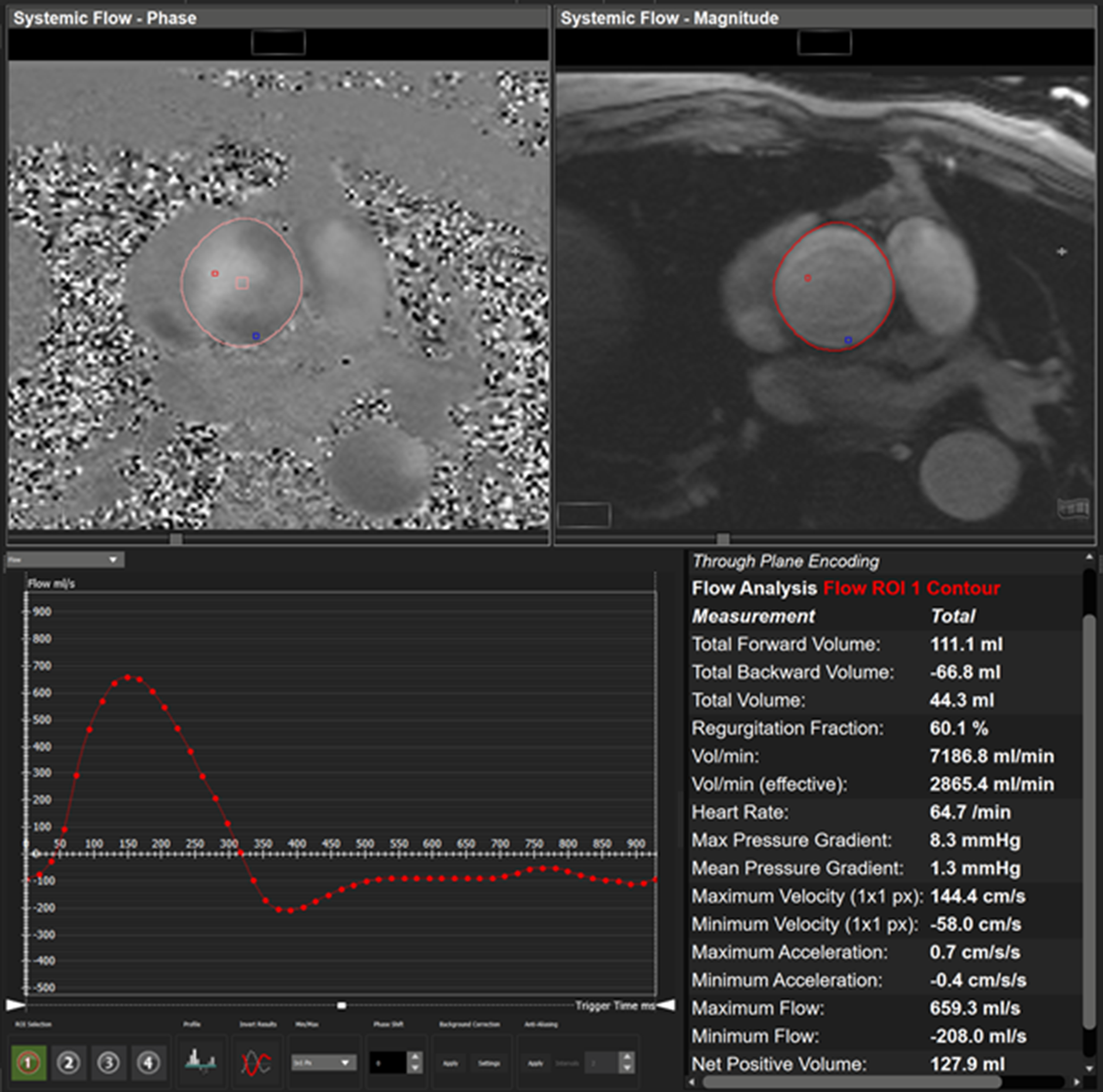

Supplement: Supplementary Figure 3 — Quantification of aortic regurgitation in the patient depicted in Figure 9 using phase-contrast velocity mapping. The magnitude image on the top right is used to contour a region of interest in the aorta. The phase image on the top left is used to determine flow and plot this against time (bottom). The flow drops below the baseline for the whole of systole. The regurgitant volume is ~67 ml which amounts to a regurgitant fraction of 60%, denoting severe regurgitation. [file Image_3.PNG]

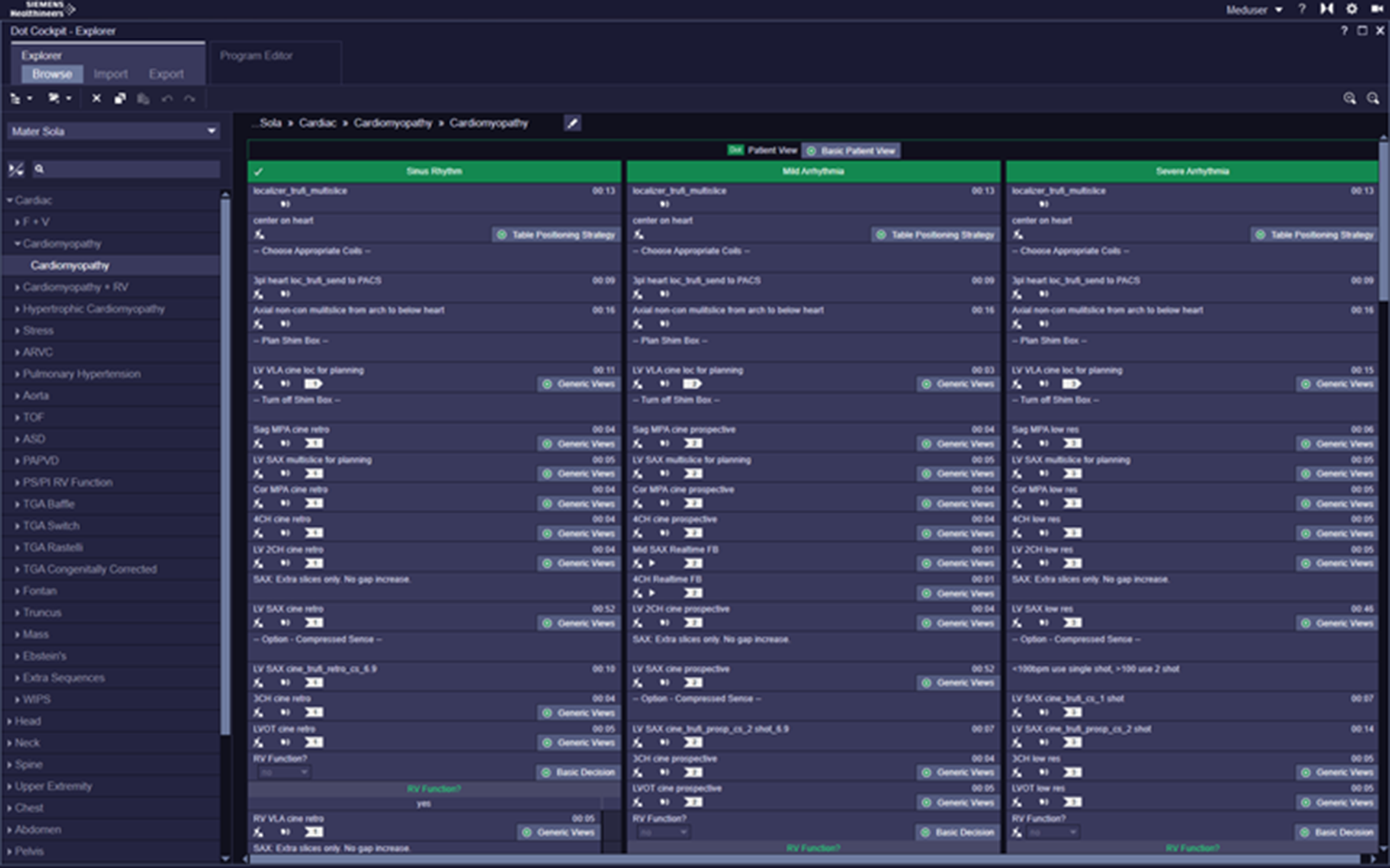

Supplement: Supplementary Figure 4 — An example of a clinical imaging protocol library built with three acquisition strategies for managing irregular rhythms: Sinus Rhythm; Mildly Irregular Rhythm; Severely Irregular Rhythm. [file Image_4.PNG]
