# Supplementary material for: Cardiac MR: From Theory to Practice
Source: Front Cardiovasc Med. 2022 Mar 3;9:826283. doi: 10.3389/fcvm.2022.826283 (PMC8927633; doi:10.3389/fcvm.2022.826283)
Supplement: Supplementary Table 1 — A general cardiomyopathy protocol can be modified to assess acute presentations by the addition of imaging sequences sensitive to oedema (highlighted). [file Table_1.DOCX]

**Acute Cardiomyopathy Protocol**

**Imaging Protocol**

1. 3-plane localiser
2. Axial non-cine bSSFP localiser series
3. LV VLA cine bSSFP localiser
4. Sagittal oblique MPA cine bSSFP
5. LVSAX multislice localiser
6. Coronal oblique MPA cine bSSFP
7. 4-chamber cine bSSFP
8. LV 2-chamber cine bSSFP
9. RV VLA cine bSSFP (if RV function required)
10. RV SAX cine bSSFP (if RV function required)
11. LV SAX cine bSSFP
12. 3-chamber cine bSSFP
13. LVOT cine bSSFP
14. LV SAX T2W fat saturated dark blood – matched to LVSAX series
15. 4-chamber T1W dark blood
16. Native T1 Map
17. T2 Map

**Inject contrast according to dose protocol**

1. Immediate LV 2-chamber LGE series (if required to exclude presence of thrombus)
2. Flow cine PC Aorta
3. Flow cine PC Aortic Valve
4. Flow cine PC MPA
5. TI Scout
6. LV SAX LGE series
7. LV 2-chamber LGE series
8. 4-chamber LGE series
